# AC Magnetic Susceptibility: Mathematical Modeling and Experimental Realization on Poly-Crystalline and Single-Crystalline High-T_c_ Superconductors YBa_2_Cu_3_O_7−δ_ and Bi_2−x_Pb_x_Sr_2_Ca_2_Cu_3_O_10+y_

**DOI:** 10.3390/ma17081744

**Published:** 2024-04-10

**Authors:** Petros Moraitis, Loukas Koutsokeras, Dimosthenis Stamopoulos

**Affiliations:** Department of Physics, School of Science, National and Kapodistrian University of Athens, Zografou Panepistimioupolis, 15784 Athens, Greece; morapet@phys.uoa.gr (P.M.);

**Keywords:** AC magnetic susceptibility, high-T_c_ superconductors, demagnetizing effect, magnetic properties

## Abstract

The multifaceted inductive technique of AC magnetic susceptibility (ACMS) provides versatile and reliable means for the investigation of the respective properties of magnetic and superconducting materials. Here, we explore, both mathematically and experimentally, the ACMS set-up, based on four coaxial pick-up coils assembled in the second-derivative configuration, when employed in the investigation of differently shaped superconducting specimens of poly-crystalline YBa_2_Cu_3_O_7−δ_ and Bi_2−x_Pb_x_Sr_2_Ca_2_Cu_3_O_10+y_ and single-crystalline YBa_2_Cu_3_O_7−δ_. Through the mathematical modeling of both the ACMS set-up and of linearly responding superconducting specimens, we obtain a closed-form relation for the DC voltage output signal. The latter is translated directly to the so-called *extrinsic* ACMS of the studied specimen. By taking into account the specific characteristics of the studied high-T_c_ specimens (such as the shape and dimensions for the demagnetizing effect, porosity for the estimation of the superconducting volume fraction, etc.), we eventually draw the truly *intrinsic* ACMS of the parent material. Importantly, this is carried out without the need for any calibration specimen. The comparison of the mathematical modeling with the experimental data of the aforementioned superconducting specimens evidences fair agreement.

## 1. Introduction

The inductive technique of AC magnetic susceptibility (ACMS) is based on the ability of an assembly of pick-up coils (PUCs) to sense temporal variations in magnetic flux [1,2]. In this respect, many different configurations of PUCs have been explored so far to inductively sense the signal of a specimen. The most popular ones are based on the coaxial adjustment of one, two and four PUCs, in the so-called zeroth-, first- and second-derivative configuration [3,4,5,6,7,8]. Due to the flexibility in the choice of PUC configuration and the relatively low-cost realization, ACMS is surely one of the most popular among the plethora of important experimental techniques used to assess the properties of magnetic [9,10,11,12,13] and superconducting [14,15,16,17,18,19,20,21] materials from room temperature down to cryogenic conditions [1]. Also, referring to dynamic phenomena, due to its inherent versatility in the frequency domain (from Hz to tens of kHz), ACMS is the technique of choice in many areas of physics and materials science used to investigate out-of-equilibrium processes such as domain wall motion and domain reversal in ferromagnets [9,10], and flux flow and creep/depinning of vortices in superconductors [1,14,16,20,21].

However, due to our longstanding empirical engagement with ACMS, we probably consider it a relatively simple technique of quite limited competence, thus underestimating its wide potential. This deceptive perception in part stems from the lack of detailed, direct modeling of ACMS hardware per se. Indeed, this is not an easy task; in almost all cases, the output signal of the experimental hardware is a DC voltage that somehow should be translated to the desired physical property of the ACMS. In principle, this should be carried out through detailed mathematical modeling that apparently is quite laborious. Thus, not surprisingly, in most of the investigations reported in the literature, ‘arbitrary units’ are used for the recorded DC voltage signal such that, as a consequence, it only has a qualitative character.

To obtain reliable quantitative insight, in practice, the DC voltage signal is usually quantified rather empirically by using a standard specimen that should exhibit a reference ACMS value, expected to hold under specific circumstances. For instance, a superconducting specimen should exhibit ideal diamagnetism in the Meissner state; under these circumstances, the recorded DC voltage signal should be attributed to an ACMS equal to −1 [14,15,22]. In the same spirit, following even more reliable procedures, a set of standard magnetic specimens of known ACMS should be employed for the direct calibration of the DC voltage signal, recorded at the output of the experimental set-up, and its reliable translation to the ACMS over a wide range of values [14,15,22]. Nevertheless, even when this is feasible, the calibration specimen should have the same shape and dimensions as the specimen under investigation, or else the demagnetizing factor will be different for the two cases, resulting in discrepancies. Due to these reasons, the reliable mathematical modeling of the ACMS technique is of paramount importance; obviously, the design of experimental set-ups with tailored specifications that will used for the quantitative assessment of the intrinsic properties of materials cannot be based on empirical guidelines.

Here, we study the ACMS technique based on the coaxial adjustment of four PUCs in the second-derivative configuration (SDC). We introduce a conceptually concise mathematical model to describe the evolution of the signal through all stages of the experimental set-up. Our model incorporates the underlying relevant physical mechanisms of linearly responding specimens that in our case refer to differently shaped specimens of two high-T_c_ superconducting materials, poly-crystalline YBa_2_Cu_3_O_7−δ_ and Bi_2−x_Pb_x_Sr_2_Ca_2_Cu_3_O_10+y_, as well as single-crystalline YBa_2_Cu_3_O_7−δ_. Importantly, our approach enables us to unveil detailed information on the middle-stage AC voltage signal, $V_{\mathrm{AC}}\left(t,T\right)$, which is delivered by the PUCs to the input of the employed Lock-In Amplifier (LIA). Eventually, from the end-stage DC voltage $V_{\mathrm{DC}}\left(T\right)$, obtained at the output of the LIA, we recover the *extrinsic* ACMS of the specimen. Most importantly, by taking into account the specific characteristics of the studied high-T_c_ specimens (that is, shape and dimensions for the demagnetizing effect, porosity for the estimation of the superconducting volume fraction, etc.), we succeed in drawing the truly *intrinsic* ACMS of the parent material, without the need for any reference specimen/material to calibrate the set-up. The comparison of the detailed mathematical model with the experimental results obtained on the aforementioned high-T_c_ materials, YBa_2_Cu_3_O_7−δ_ and Bi_2−x_Pb_x_Sr_2_Ca_2_Cu_3_O_10+y_, evidences fair quantitative agreement.

## 2. Experimental Techniques and Materials

### 2.1. ACMS Experimental Set-Up

**Figure 1:**
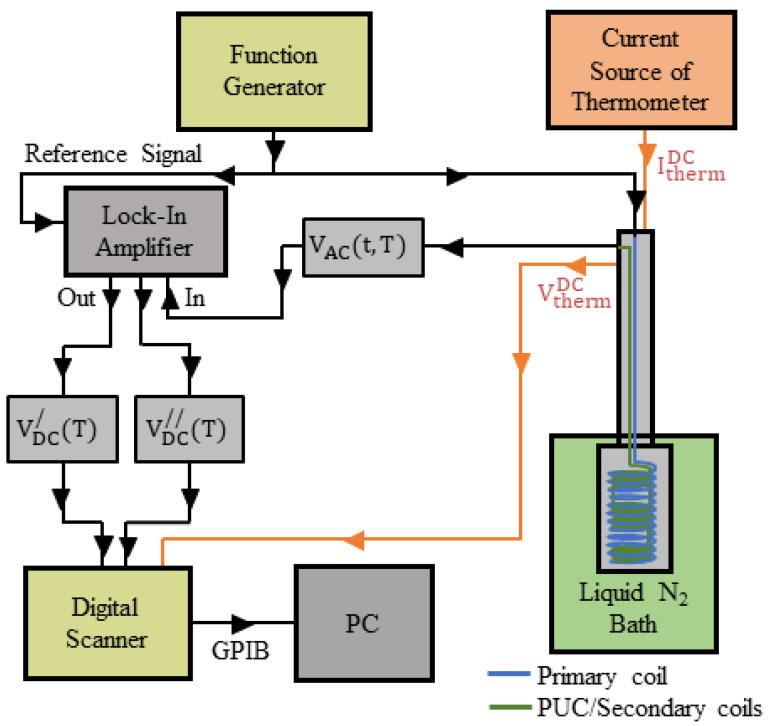
Schematic diagram of the components of the ACMS experimental set-up employed in our laboratory. The unit is divided into five main parts: (i) A function generator that drives the primary coil for the production of the external, harmonic, uniform AC magnetic field, $H_{\mathrm{ext}}\left(r,t\right)=H_{0}\cos\left(\omega t\right)\hat{z}$. (ii) The assembly of PUCs in the SDC which inductively records the AC voltage signal, $V_{\mathrm{AC}}\left(t,T\right)$, induced by the alternating magnetization, $M_{\mathrm{AC}}\left(r,t,T\right)$, of the specimen hosted inside the probe (see Figure 2, below), in response to $H_{\mathrm{ext}}\left(r,t\right)$. (iii) The probe which hosts the primary/secondary coils and the specimen. (iv) The Lock-In Amplifier that amplifies the input signal, $V_{\mathrm{AC}}\left(t,T\right)$, eventually providing, at its two outputs, the DC voltages: real $V_{\mathrm{DC}}^{/}\left(T\right)$ (Relation (1)) and imaginary $V_{\mathrm{DC}}^{//}\left(T\right)$ (Relation (2)). (v) The Digital Scanner assists the PC in recording both signals, $V_{\mathrm{DC}}^{/}\left(T\right)$ and $V_{\mathrm{DC}}^{//}\left(T\right)$, by using a General Purpose Interface Bus (GPIB) connection.

**Figure 2:**
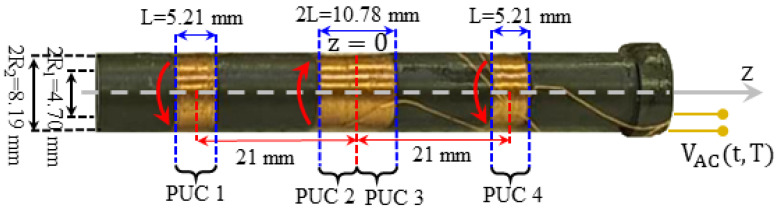
Assembly of four coaxial PUCs, 1, 2, 3 and 4, combined in the SDC to detect the AC voltage signal, $V_{\mathrm{AC}}\left(t,T\right)$, induced by the alternating magnetization of the specimen, $M_{\mathrm{AC}}\left(r,t,T\right)$, in response to an external, harmonic, uniform AC magnetic field, $H_{\mathrm{ext}}\left(r,t\right)=H_{0}\cos\left(\omega t\right)\hat{z}$. The outer PUCs 1 and 4 are single, while the middle PUCs 2 and 3 form a double coil centered at z=0. The PUCs are assembled on an insulating, hollow cylindrical holder that hosts the sample, placed at the center of PUCs 2 and 3 (z=0). PUCs 1 and 4 (outer coils) have the same winding direction, opposite to that of 2 and 3 (inner coils), as shown by the red arrows. This ensures that the assembly is not excited by a uniform nor by a linearly varying external magnetic field. All important dimensions of each PUC and of their relative positions are shown. See text for details.

The most common mode of operation of an ACMS experimental set-up is to record an AC voltage signal, $V_{\mathrm{AC}}\left(t,T\right)$, which closely relates to the alternating magnetization of a specimen subjected to an external AC magnetic field, while varying the temperature, T. The respective home-made experimental set-up employed in our laboratory is shown schematically in Figure 1. To record the $V_{\mathrm{AC}}\left(t,T\right)$, we employ an assembly of four coaxial PUCs in the SDC [3,4,5,6,7,8], as shown in Figure 2. The PUCs have their surface perpendicular to the z-axis and they are placed symmetrically in respect to z=0. In addition, in the SDC, the outer PUCs 1 and 4 have the same winding direction that is opposite to that of the inner PUCs 2 and 3. The SDC ensures that the four coaxial PUCs are not excited by a uniform nor by a linearly varying external magnetic field. The specimen is placed at the center of the middle, double coil, which is at z=0 and is subjected to the external, harmonic, uniform AC magnetic field, $H_{\mathrm{ext}}\left(r,t\right)=H_{0}\cos\left(\omega t\right)\hat{z}$. The alternating magnetization of the specimen, $M_{\mathrm{AC}}\left(r,t,T\right)$, will induce an alternating magnetic flux in the assembly of PUCs that in turn will induce a relatively weak AC voltage signal, $V_{\mathrm{AC}}\left(t,T\right)$. The selective amplification of $V_{\mathrm{AC}}\left(t,T\right)$, is performed by an LIA (SR530, Stanford Research Systems, Sunnyvale, CA, USA) empowered with a voltage gain factor up to 10^9^ (sensitivity down to 10^−9^). The LIA ultimately provides an end-stage DC voltage signal, $V_{\mathrm{DC}}\left(T\right)$ [23], as an output, which relates to the temperature variation of the ACMS of the specimen. This DC voltage signal, $V_{\mathrm{DC}}\left(T\right)$, is provided in two forms, the so-called in-phase/real/cosinusoidal, $V_{\mathrm{DC}}^{/}\left(T\right)$, and out-of-phase/imaginary/sinusoidal, $V_{\mathrm{DC}}^{//}\left(T\right)$, at the two distinct outputs of the LIA:

(1) $$V_{\mathrm{DC}}^{/}\left(T\right)=V_{\mathrm{AC},0}\left(T\right)\;\frac{\cos\theta }{\sqrt{2}}\frac{10\;V}{\mathrm{sensitivity}}$$

(2) $$V_{\mathrm{DC}}^{//}\left(T\right)=V_{\mathrm{AC},0}\left(T\right)\;\frac{\sin\theta }{\sqrt{2}}\frac{10\;V}{\mathrm{sensitivity}}$$

In these relations: (i) $V_{\mathrm{AC},0}\left(T\right)$, is the amplitude of the middle-stage AC voltage signal, $V_{\mathrm{AC}}\left(t,T\right)$. (ii) θ is the overall phase/angle that appears at the two output DC voltage signals. Specifically, θ should be adjusted to the right value by an additional relative phase/angle, θ_LIA_, provided by the LIA, so that the two output DC voltage signals, $V_{\mathrm{DC}}^{/}\left(T\right)$ and $V_{\mathrm{DC}}^{//}\left(T\right)$, conform to the physics of the studied specimen. (iii) ‘Sensitivity^−1^′ is actually the voltage gain factor that can be selectively applied to the input signal, $V_{\mathrm{AC}}\left(t,T\right)$, at the desired frequency of the ‘Reference Signal’, and (iv) ‘10 V’ is the maximum value of the output DC voltage signals, $V_{\mathrm{DC}}^{/}\left(T\right)$ and $V_{\mathrm{DC}}^{//}\left(T\right)$ (see the analytical discussion below in Section 3). (v) Finally, in the above Relations (1) and (2), an ‘Offset’ voltage that can appear in general and an additional amplification factor ‘Expand’ (that equals 10 or 100) have been omitted, since they do not influence the basic operation of the LIA and the mathematical model presented in this work.

Eventually, both output signals, $V_{\mathrm{DC}}^{/}\left(T\right)$ and $V_{\mathrm{DC}}^{//}\left(T\right)$, are recorded by a PC, in our case through a Digital Scanner (Keithley DM2000, Solon, OH, USA). The recorded signals, $V_{\mathrm{DC}}^{/}\left(T\right)$ and $V_{\mathrm{DC}}^{//}\left(T\right)$, are closely related to the so-called *extrinsic* ACMS, $\chi_{m,\mathrm{AC}}^{\mathrm{ex}}\left(T\right)$ (else, *as-measured* ACMS), of the specimen under investigation. It should be noted that $\chi_{m,\mathrm{AC}}^{\mathrm{ex}}\left(T\right)$ depends on (i) the shape and dimensions of each particular specimen and (ii) the configuration of the externally applied magnetic field with respect to the surfaces of the particular specimen. These factors determine the degree of contribution of demagnetizing effects that are inevitably always present in specimens of finite size [22,24,25,26,27,28]. Due to these reasons, $V_{\mathrm{DC}}^{/}\left(T\right)$ and $V_{\mathrm{DC}}^{//}\left(T\right)$ cannot directly provide the truly *intrinsic* ACMS, $\chi_{m,\mathrm{AC}}^{\mathrm{in}}\left(T\right)$, of the parent material [22,27,28]. To recover the $\chi_{m,\mathrm{AC}}^{\mathrm{in}}\left(T\right)$ of the parent material from the $\chi_{m,\mathrm{AC}}^{\mathrm{ex}}\left(T\right)$ of the particular specimen, $V_{\mathrm{DC}}^{/}\left(T\right)$ and $V_{\mathrm{DC}}^{//}\left(T\right)$ should be processed on the basis of a suitable mathematical model that properly incorporates the underlying physics. To this end, demagnetizing effects can be taken into account either directly/analytically or indirectly/computationally, depending on the difficulties of the algebraic calculations (see the analytical discussion below in Section 4).

Figure 2 shows the assembly of the four nominally identical, coaxial PUCs combined in the SDC. Each PUC has the same number of turns, N (typically, 500≤N≤700; here, ~675), made of thin copper wire (typically, 0.05 mm ≤ thickness ≤ 0.20 mm; here, 0.12 mm). PUCs 1 and 4 (outer coils) have the same winding direction, opposite to that of 2 and 3 (inner coils). This ensures that the assembly is not excited by a uniform nor by a linearly varying external magnetic field. Thus, the PUCs in the SDC solely detect the AC voltage signal, $V_{\mathrm{AC}}\left(t,T\right)$, induced by the alternating magnetization of the specimen, $M_{\mathrm{AC}}\left(r,t,T\right)$, in response to an external, harmonic, uniform AC magnetic field, $H_{\mathrm{ext}}\left(r,t\right)=H_{0}\cos\left(\omega t\right)\hat{z}$. The outer PUCs 1 and 4 are single, while the middle PUCs 2 and 3 form a double coil centered at z=0. The PUCs are assembled on an insulating, hollow cylindrical holder of outer diameter $2R_{2}=$ 8.19 mm and inner diameter $2R_{1}=$ 4.70 mm. The specimen is placed at the center of PUCs 2 and 3 (z=0) so that maximum magnetic flux is recorded (see Section 3, below).

In our home-made unit, we can perform measurements with an excitation AC magnetic field of amplitude (rms value) $0.01\;G\leq B_{\mathrm{AC}}^{\mathrm{rms}}\leq 2G$ and frequency $1\;\mathrm{Hz}\leq f_{\mathrm{AC}}\leq 10\;\mathrm{kHz}$. At the same time, if needed, we can apply a DC magnetic field, $-500\;G\leq B_{\mathrm{DC}}\leq 500\;G$. The experimental set-up operates in the temperature range 78 K≤T≤298 K. The sensitivity of the assembly of coaxial PUCs in the SDC is very high. Specifically, for a superconducting reference specimen of *intrinsic* ACMS, $\chi_{m}=-1$ (perfect diamagnetism, Meissner state), it is greater than $1\;\mu V/\left(\mathrm{mg}\cdot G\right)$. This, combined with the use of the LIA, allows us to measure specimens with a mass of less than 1 mg (see the detailed discussion below in Section 5.3). This can be particularly important for materials that cannot be produced in large quantities.

### 2.2. X-ray Diffractometer

The crystal structure and the phase purity of the poly-crystalline Bi_2−x_Pb_x_Sr_2_Ca_2_Cu_3_O_10+y_ and YBa_2_Cu_3_O_7−δ_ superconducting specimens were investigated by the X-ray Diffraction (XRD) method. The powdered samples have been measured in a X-ray diffractometer (Siemens D5000, Dallas, TX, USA), equipped with a Cu tube and a graphite monochromator in receiving optics. All patterns were collected in Bragg–Brentano scans without sample rotation.

### 2.3. Scanning Electron Microscopy

The microstructure of the poly-crystalline Bi_2−x_Pb_x_Sr_2_Ca_2_Cu_3_O_10+y_ and YBa_2_Cu_3_O_7−δ_ superconducting specimens was evaluated by Scanning Electron Microscopy (SEM) using a thermal emission microscope (Quanta 200, FEI Technologies Inc., Hillsboro, OR, USA). The samples were mounted on aluminum stubs with conductive adhesive tape and examined without any further coating. The SEM images were acquired in the Secondary Electron Detection mode of operation, at 20 kV accelerating voltage and at working distances within 8 and 12 mm.

### 2.4. Materials

In this work, we study differently shaped specimens of two superconducting materials, poly-crystalline YBa_2_Cu_3_O_7−δ_ and Bi_2−x_Pb_x_Sr_2_Ca_2_Cu_3_O_10+y_. We also investigate the highly demanding case of single-crystalline YBa_2_Cu_3_O_7−δ_. The poly-crystalline bulk samples were prepared by means of standard methods of solid-state chemistry.

The starting materials were weighed by means of a high-precision digital balance of four decimal points (Explorer Analytical Balance, Ohaus, Parsippany-Troy Hills, NJ, USA), then they were carefully mixed/homogenized manually for at least 15 min by using an Agate pestle and mortar and finally shaped into the desired cylinder/disc by means of an appropriate die of stainless steel (diameter 4.6 mm) under application of a pressure of 100 bar for at least 1 min by using a hydraulic press. Then, the compacted samples were transferred to a crucible made of alumina (Al_2_O_3_) and placed inside a laboratory furnace (TZF 12/65/550, Carbolite-Gero Ltd., Hope Rd, UK) for sintering at the desired conditions (i.e., temperature ramp rate, maximum temperature, duration, etc.).

Poly-crystalline YBa_2_Cu_3_O_7−δ_: For the production of poly-crystalline YBa_2_Cu_3_O_7−δ_, stochiometric quantities of the following chemical reactants were used: $Y_{2}O_{3}$ (Sigma-Aldrich, St. Louis, MO, USA, purity 99.99%), ${\mathrm{BaCO}}_{3}$ (Alfa Aesar, Ward Hill, MA, USA, purity 99.95%) and CuO (Alfa Aesar, purity 99.70%) following the reaction

$$1/2Y_{2}O_{3}+2{\mathrm{BaCO}}_{3}+3\mathrm{CuO}\to {\mathrm{YBa}}_{2}{\mathrm{Cu}}_{3}O_{7-\delta }+2{\mathrm{CO}}_{2}$$

The materials were sintered at 920 °C (ramp rate 5 °C/min) for 24 h and then left to cool down to room temperature. This process provides the desired oxygen content, 0.05≤δ≤0.10, which relates to the maximum critical temperature T_c_ ≈ 93 K [29,30,31,32,33,34].

Poly-crystalline Bi_1.6_Pb_0.4_Sr_1.6_Ca_2.0_Cu_2.8_O_9.2+x_: For the production of poly-crystalline Bi_2−x_Pb_x_Sr_2−y_Ca_2−z_Cu_3−w_O_10−δ_, we employed a commercially available relevant chemical compound with the nominal composition Bi_1_._6_Pb_0_._4_Sr_1_._6_Ca_2_._0_Cu_2_._8_O_9_._2_ (Sigma-Aldrich) that was sintered at 845 °C (ramp rate 5 °C/min) for 24 h and then left to cool down to room temperature. This process preserves the desired Pb and O contents, x≈0.4 and δ≈0.8, respectively, which relate to the maximum critical temperature T_c_ ≈ 110 K [35,36,37,38,39].

Single-crystalline YBa_2_Cu_3_O_7−δ_: For the production of single-crystalline YBa_2_Cu_3_O_7−δ_, non-stoichiometric quantities of the following chemical reactants were used: $Y_{2}O_{3}$ (Sigma-Aldrich, purity 99.99%), ${\mathrm{BaCO}}_{3}$ (Alfa Aesar, purity 99.95%) and CuO (Alfa Aesar, purity 99.70%); the following reaction applies only loosely:

$$1/2Y_{2}O_{3}+2{\mathrm{BaCO}}_{3}+3\mathrm{CuO}\to {\mathrm{YBa}}_{2}{\mathrm{Cu}}_{3}O_{7-\delta }+2{\mathrm{CO}}_{2}$$

After homogenization, the material was placed inside a Au crucible and sintered at a temperature slightly above the melting point (>920 °C) for a duration of up to a few days. Then, it was subjected to slow cooling to room temperature. Whenever needed, further processing was performed at 450 °C up to a few weeks to obtain the optimum oxygen content 0.05≤δ≤0.10 that relates to the maximum critical temperature T_c_ ≈ 93 K [29,30,31,32,33,34].

## 3. Mathematical Modeling of the ACMS Experimental Set-Up

Here, we present a detailed mathematical model of the ACMS experimental set-up, which comprises four coaxial PUCs in the SDC, in combination with an LIA. First, let us consider the basic case of a linear and isotropic, however inhomogeneous, magnetic or superconducting specimen subjected to an externally applied, uniform, harmonic AC magnetic field, $H_{\mathrm{ext}}\left(r,t\right)=H_{0}\cos\left(\omega t\right)\hat{z}$. The specimen, through its scalar ACMS, $\chi_{m,\mathrm{AC}}\left(r,T\right)$, will develop an alternating magnetization, $M_{\mathrm{AC}}\left(r,t,T\right)=M_{\mathrm{AC}}\left(r,t,T\right)\hat{z}$ (at the moment, we do not determine whether $\chi_{m,\mathrm{AC}}\left(r,T\right)$ refers to the *extrinsic* $\chi_{m,\mathrm{AC}}^{\mathrm{ex}}\left(T\right)$ or to the *intrinsic* $\chi_{m,\mathrm{AC}}^{\mathrm{in}}\left(T\right)$ ACMS). The PUCs are not excited by the uniform $H_{\mathrm{ext}}\left(r,t\right)$, due to the SDC [3,4,5,6,7,8]. On the contrary, they inductively produce an AC voltage signal, $V_{\mathrm{AC}}\left(t,T\right)$, in response to the time variation of the magnetic flux that they sense due to the alternating magnetization of the specimen, $M_{\mathrm{AC}}\left(r,t,T\right)=M_{\mathrm{AC}}\left(r,t,T\right)\hat{z}$. To facilitate the algebraic part and to obtain closed-form relations that are useful for a straightforward comparison with experimental data, the specimen is almost always treated as an ideal point-like magnetic dipole. Accordingly, the magnetic dipole moment of the specimen should be obtained through the basic relation

(3) $$m_{\mathrm{AC}}\left(t,T\right)=m_{\mathrm{AC}}\left(t,T\right)\hat{z}=\int_{V}^{\;}M_{\mathrm{AC}}\left(r,t,T\right)\mathrm{dV}\hat{z}$$

given that $M_{\mathrm{AC}}\left(r,t,T\right)=M_{\mathrm{AC}}\left(r,t,T\right)\hat{z}$ has been calculated beforehand, by analytically or computationally solving the respective electromagnetic problem, a linear and isotropic, however inhomogeneous, specimen of ACMS, $\chi_{m,\mathrm{AC}}\left(r,T\right)$, is subjected to a uniform magnetic field, $H_{\mathrm{ext}}\left(r,t\right)=H_{0}\cos\left(\omega t\right)\hat{z}$, so that it develops an inhomogeneous magnetization, $M_{\mathrm{AC}}\left(r,t,T\right)=M_{\mathrm{AC}}\left(r,t,T\right)\hat{z}$. In this way, the $m_{\mathrm{AC}}\left(t,T\right)$, obtained through Relation (3), carries all the underlying information on the magnetic properties (ACMS, $\chi_{m,\mathrm{AC}}\left(r,T\right)$) of the specimen. Based on Faraday’s law, $V_{\mathrm{AC}}\left(t,T\right)=-d\Phi \left(t,T\right)/\mathrm{dt}$, it follows that the middle-stage AC voltage signal, $V_{\mathrm{AC}}\left(t,T\right)$, induced at the assembly of the four PUCs by the alternating moment, $m_{\mathrm{AC}}\left(t,T\right)$, of the point-like magnetic dipole/specimen is given by the following relation [3]:

(4) $$V_{\mathrm{AC}}\left(t,T\right)=-\mu_{0}\frac{{\mathrm{dm}}_{\mathrm{AC}}\left(t,T\right)}{\mathrm{dt}}F_{\mathrm{PUC}-\mathrm{SDC}}$$

Notice that since $\chi_{m,\mathrm{AC}}\left(r,T\right)$, $M_{\mathrm{AC}}\left(r,t,T\right)$ and $m_{\mathrm{AC}}\left(t,T\right)$ all depend on temperature, the middle-stage AC voltage signal, $V_{\mathrm{AC}}\left(t,T\right)$, of the PUCs should be a function of the specimen’s temperature, T, as well. Indeed, this is evidenced in the above Relation (4). Also, $F_{\mathrm{PUC}-\mathrm{SDC}}$ is the so-called Sensing Function of the PCUs in the SDC that is discussed in detail below. At the moment, we underline that due to the linear character of the underlying physics studied here, Relation (4) can be rewritten as [3]

(5) $$V_{\mathrm{AC}}\left(t,T\right)=V_{\mathrm{AC},0}\left(T\right)V_{\mathrm{AC}}\left(\omega t\right)=-\mu_{0}{\left(\frac{{\mathrm{dm}}_{\mathrm{AC}}\left(t,T\right)}{\mathrm{dt}}\right)}_{0}\frac{{\mathrm{dm}}_{\mathrm{AC}}\left(\omega t\right)}{\mathrm{dt}}F_{\mathrm{PUC}-\mathrm{SDC}}$$

Here, the functions $V_{\mathrm{AC}}\left(t,T\right)$ and ${\mathrm{dm}}_{\mathrm{AC}}\left(t,T\right)/\mathrm{dt}$ are explicitly decomposed in a separation-of-variables scheme, where $V_{\mathrm{AC},0}\left(T\right)$ and ${\left({\mathrm{dm}}_{\mathrm{AC}}\left(t,T\right)/\mathrm{dt}\right)}_{0}$ are their time-independent, temperature-dependent amplitudes and $V_{\mathrm{AC}}\left(\omega t\right)$ and ${\mathrm{dm}}_{\mathrm{AC}}\left(\omega t\right)/\mathrm{dt}$ are their time-dependent, temperature-independent, dimensionless parts. These entities should be calculated for each specific problem under investigation (see Section 4, ‘Superconducting cylinder—Complete mathematical modeling of the ACMS’, below). Returning to the so-called Sensing Function, $F_{\mathrm{PUC}-\mathrm{SDC}}$, we note that it quantitatively expresses a specific assembly of four coaxial PUCs that can ‘translate’ the time variation of the alternating moment, $m_{\mathrm{AC}}\left(t,T\right)$, of a point-like magnetic dipole/specimen into an AC signal, $V_{\mathrm{AC}}\left(t,T\right)$. Reasonably, the Sensing Function, $F_{\mathrm{PUC}-\mathrm{SDC}}$, should depend solely on the characteristics (i) of each secondary coil in particular (such as number of turns, inner/outer diameter, thickness, length, etc.) and (ii) of the assembly of the coaxial PUCs in general (such as distance between the secondary coils, etc.). After relatively extensive algebraic calculations (this purely algebraic part will be discussed elsewhere), it is shown that the magnetic flux, $\Phi_{\mathrm{PUC}-\mathrm{SDC}}^{\;}\left(t,T\right)$, recorded by the assembly of the four coaxial PUCs in the SDC, is maximum when the specimen is positioned at the center of the middle, double PUC, which is at z=0 (see Figure 2, above). The respective maximum value, $\Phi_{\mathrm{PUC}-\mathrm{SDC}}^{\max}\left(t,T\right)$, is given by the relation [3]

(6) $$\Phi_{\mathrm{PUC}-\mathrm{SDC}}^{\max}\left(t,T\right)=\mu_{0}m_{\mathrm{AC}}\left(t,T\right)\frac{N}{\mathrm{LD}}\left[L\ln\left[\frac{R_{2}+\sqrt{R_{2}^{2}+L^{2}}}{R_{1}+\sqrt{R_{1}^{2}+L^{2}}}\right]+\left(z_{c1}-\frac{L}{2}\right)\ln\left[\frac{R_{2}+\sqrt{R_{2}^{2}+{\left(z_{c1}-\frac{L}{2}\right)}^{2}}}{R_{1}+\sqrt{R_{1}^{2}+{\left(z_{c1}-\frac{L}{2}\right)}^{2}}}\right]\;\right.\;\left.+\left(z_{c4}-\frac{L}{2}\right)\ln\left[\frac{R_{2}+\sqrt{R_{2}^{2}+{\left(z_{c4}-\frac{L}{2}\right)}^{2}}}{R_{1}+\sqrt{R_{1}^{2}+{\left(z_{c4}-\frac{L}{2}\right)}^{2}}}\right]\;\right]$$

Here, N, $R_{1}$, $R_{2}$, $D=R_{2}-R_{1}$ and L refer to the total number of turns, the inner radius, the outer radius, and the thickness and the length of each identical PUC, respectively. Also, $z_{c1}$ and $z_{c4}$ refer to the z-axis position of the center of the outer PUCs 1 and 4, respectively. These parameters are illustrated in Figure 2, above. We should note that the first, second and third term in parentheses correspond to the contribution of the middle/double, first and fourth PUCs, respectively. Extensive investigations of Relation (6) through simulations in the realistic range of parameters employed in standard experimental set-ups (500≤N≤700, $4.5\;\mathrm{mm}\leq R_{1}\leq 5.0\;\mathrm{mm}$, $7.0\;\mathrm{mm}\leq R_{2}\leq 9.0\;\mathrm{mm}$, 4.5 mm≤L≤5.5 mm and $1.5\;\mathrm{mm}\leq \left|z_{c1}\right|,{\;z}_{c4}\leq 2.5\;\mathrm{mm}$) evidenced that the contribution of the second and third terms of the parentheses (first and fourth PUCs of the assembly shown in Figure 2) are below 1% (specifically, in the order of 0.7%). Thus, these terms can be rightfully neglected so that Relation (6) obtains a more convenient form [3]:

(7) $$\Phi_{\mathrm{PUC}-\mathrm{SDC}}^{\max}\left(t,T\right)=\mu_{0}m_{\mathrm{AC}}\left(t,T\right)\frac{N}{D}\ln\left[\frac{R_{2}+\sqrt{R_{2}^{2}+L^{2}}}{R_{1}+\sqrt{R_{1}^{2}+L^{2}}}\right]$$

From this relation, we can now define the Sensing Function, $F_{\mathrm{PUC}-\mathrm{SDC}}$, of the assembly of PUCs in the SDC employed in our experimental set-up. Indeed, by combining Faraday’s law, $V_{\mathrm{AC}}\left(t,T\right)=-d\Phi \left(t,T\right)/\mathrm{dt}$, with Relations (4) and (7), we define $F_{\mathrm{PUC}-\mathrm{SDC}}$ through the relation [3]

(8) $$F_{\mathrm{PUC}-\mathrm{SDC}}=\frac{N}{D}\ln\left[\frac{R_{2}+\sqrt{R_{2}^{2}+L^{2}}}{R_{1}+\sqrt{R_{1}^{2}+L^{2}}}\right]$$

Now, we are able to proceed to the final stage of our experimental set-up where the inductive middle-stage AC voltage signal, $V_{\mathrm{AC}}\left(t,T\right)$, of Relations (4) and (5) is supplied to the input of the LIA (see Figure 1). Before doing this, let us recall the basics in the operation of an LIA for the general case. In brief, an LIA performs as follows: (i) It isolates the desired AC component, $V_{\mathrm{AC}}\left(t\right)$, at a reference angular frequency, ω, from a highly noisy, multi-frequency, input voltage signal. (ii) It selectively amplifies $V_{\mathrm{AC}}\left(t\right)$ with a high gain factor (sensitivity^−1^) up to 10^9^ (or even higher). (iii) It provides two output DC voltage signals, the in-phase/real/cosinusoidal, $V_{\mathrm{DC}}^{/\;}$, and the out-of-phase/imaginary/sinusoidal, $V_{\mathrm{DC}}^{//\;}$. Both signals, $V_{\mathrm{DC}}^{/\;}$ and $V_{\mathrm{DC}}^{//\;}$, are proportional to the rms value, $V_{\mathrm{AC}}^{\mathrm{rms}}$ (else, to the amplitude, $V_{\mathrm{AC},0}=\sqrt{2}V_{\mathrm{AC}}^{\mathrm{rms}}$) of the AC input signal, $V_{\mathrm{AC}}\left(t\right)$, up to a maximum value that in most cases is 10 V, thus $V_{\mathrm{DC}}^{/\;},\;V_{\mathrm{DC}}^{//\;}\leq 10\;V$. (iv) Finally, the LIA provides an internal ‘degree-of-freedom’, a relative phase/angle that appears in both trigonometric coefficients, cosinusoidal and sinusoidal, and enables us to selectively adjust the respective output signals, $V_{\mathrm{DC}}^{/\;}$ and $V_{\mathrm{DC}}^{//\;}$, on a comparative basis. *This facility of the LIA is very important since it enables the experienced user to unveil the underlying physics of the studied system by ascribing the proper content, both quantitatively and qualitatively, to the output signals,* $V_{DC}^{/}$ *and* $V_{DC}^{//}$ [23]. The entire process discussed above can be represented by a Transfer Function for each one of its two outputs, given by the following relations [23]:

(9) $$F_{\mathrm{LIA}}^{/}=\frac{V_{\mathrm{DC}}^{/}}{V_{\mathrm{AC}}^{\mathrm{rms}}}=\cos\theta \frac{10\;V}{\mathrm{sensitivity}}$$

(10) $$F_{\mathrm{LIA}}^{//}=\frac{V_{\mathrm{DC}}^{//}}{V_{\mathrm{AC}}^{\mathrm{rms}}}=\sin\theta \frac{10\;V}{\mathrm{sensitivity}}$$

Returning to our case, by using the amplitude $V_{\mathrm{AC},0}\left(T\right)$ of the AC signal, $V_{\mathrm{AC}}\left(t,T\right)$, induced to the assembly of PUCs in the SDC (see Relation (5)), we obtain $F_{\mathrm{LIA}}^{/}=V_{\mathrm{DC}}^{/}\left(T\right)/V_{\mathrm{AC}}^{\mathrm{rms}}\left(T\right)={\;V}_{\mathrm{DC}}^{/}\left(T\right)/\left(V_{\mathrm{AC},0}\left(T\right)/\sqrt{2}\right)$ and $F_{\mathrm{LIA}}^{//}=V_{\mathrm{DC}}^{//}\left(T\right)/V_{\mathrm{AC}}^{\mathrm{rms}}\left(T\right)=V_{\mathrm{DC}}^{//}\left(T\right)/\left(V_{\mathrm{AC},0}\left(T\right)/\sqrt{2}\right)$, so that the LIA gives two end-stage DC voltage signals at its two outputs, the in-phase/real/cosinusoidal and the out-of-phase/imaginary/sinusoidal, expressed by the following relations [3]:

(11) $$V_{\mathrm{DC}}^{/}\left(T\right)=V_{\mathrm{AC},0}\left(T\right)\;\frac{\cos\theta }{\sqrt{2}}\frac{10\;V}{\mathrm{sensitivity}}$$

(12) $$V_{\mathrm{DC}}^{//}\left(T\right)={\;V}_{\mathrm{AC},0}\left(T\right)\frac{\sin\theta }{\sqrt{2}}\frac{10\;V}{\mathrm{sensitivity}}$$

These output DC voltages depend on the temperature, T, of the specimen and are recorded automatically by the PC through a Digital Scanner (see Figure 1, above). We underline that in these expressions, $V_{\mathrm{AC},0}\left(T\right)$ is the amplitude (else, $V_{\mathrm{AC},0}\left(T\right)/\sqrt{2}=V_{\mathrm{AC}}^{\mathrm{rms}}\left(T\right)$, the rms value) of the inductive middle-stage AC voltage signal, $V_{\mathrm{AC}}\left(t,T\right)$, which appears at the output of the assembly of the four coaxial PUCs in the SDC. By recalling that $V_{\mathrm{AC}}\left(t,T\right)$ carries the information about the ACMS of the specimen (Relations (4) and (5)), we understand that the two end-stage DC voltages, $V_{\mathrm{DC}}^{/}\left(T\right)$ and $V_{\mathrm{DC}}^{//}\left(T\right)$, contain the desired information about the temperature variation of the ACMS of the specimen. Nevertheless, further mathematical modeling is needed on the basis of the underlying physics to unveil the desired information (see below).

Now, we can combine Relations (4), (5), (8), (9), (10), (11) and (12) to obtain the following compact form for the temperature-dependent, end-stage DC voltage signals provided by the LIA at its respective outputs:

(13) $$V_{\mathrm{DC}}^{/}\left(T\right)=-\mu_{0}{\left(\frac{{\mathrm{dm}}_{\mathrm{AC}}\left(t,T\right)}{\mathrm{dt}}\right)}_{0}\times F_{\mathrm{PUC}-\mathrm{SDC}}\times F_{\mathrm{LIA}}^{/}$$

and

(14) $$V_{\mathrm{DC}}^{//}\left(T\right)=-\mu_{0}{\left(\frac{{\mathrm{dm}}_{\mathrm{AC}}\left(t,T\right)}{\mathrm{dt}}\right)}_{0}\times F_{\mathrm{PUC}-\mathrm{SDC}}\times F_{\mathrm{LIA}}^{//}$$

where ${\left({\mathrm{dm}}_{\mathrm{AC}}\left(t,T\right)/\mathrm{dt}\right)}_{0}$ is the amplitude of the time derivative of the alternating magnetic dipole moment, ${\mathrm{dm}}_{\mathrm{AC}}\left(t,T\right)/\mathrm{dt}$, which the specimen develops under the excitation of the externally applied, uniform, harmonic AC magnetic field, $H_{\mathrm{ext}}\left(r,t\right)=H_{0}\cos\left(\omega t\right)\hat{z}$. *It is worth emphasizing that in the above expression, the functions* $F_{PUC-SDC}$*,* $F_{LIA}^{/}$ *and* $F_{LIA}^{//}$ *depend solely on the intrinsic characteristics of the experimental set-up; that is, they do not depend on the characteristics of the respective specimen/material. Thus, the above expressions are generic and apply to any specimen/material for which we can analytically or computationally calculate the parameter* ${\left(dm_{AC}\left(t,T\right)/dt\right)}_{0}$.

Thus, generally, the alternating dipole moment $m_{\mathrm{AC}}\left(t,T\right)$ of the particular specimen under investigation should be obtained through Relation (3). This is not so easy since every particular specimen has different characteristics of both extrinsic and intrinsic origin, such as shape, dimensions and magnetic susceptibility, $\chi_{m}\left(r,T\right)$. In the following sections, we present these issues in detail for the case of a bulk cylinder/disc of poly-crystalline superconducting YBa_2_Cu_3_O_7−δ_ and Bi_2−x_Pb_x_Sr_2_Ca_2_Cu_3_O_10+y_ and a thin plate of single-crystalline superconducting YBa_2_Cu_3_O_7−δ_. Specifically, in Section 4, we present the analytical calculations for the case of a superconducting bulk cylinder/disc, while in Section 5, ‘Experimental results’, we show representative experimental ACMS data and provide a comparative discussion with the theoretical predictions.

## 4. Superconducting Cylinder—Complete Mathematical Modeling of the ACMS

Here, we apply detailed analytical calculations for the case of a superconducting cylinder of radius a and infinite length, coaxial to the z-axis, which is subjected to an externally applied, uniform, harmonic AC magnetic field, parallel to its axis, $H_{\mathrm{ext}}\left(r,t\right)=H_{0}\cos\left(\omega t\right)\hat{z}$, with amplitude $H_{0}$, smaller than the lower critical field, $H_{c1}\left(T\right)$ (Meissner state). This case can be treated analytically due to the invariance in both translations along and rotations about the z-axis, and the absence of demagnetizing effects. Once we treat this case of infinite length, demagnetizing effects that appear in realistic specimens of finite length are taken into account indirectly through comparison with computational calculations from the literature (see below).

Returning to the specimen of infinite length, first, we have to analytically solve the electromagnetic problem, based on Maxwell equations accompanied by the London one [40,41,42].

(15) $$\nabla^{2}B_{\mathrm{tot}}\left(r,t,T\right)-\frac{1}{\lambda_{L}^{2}\left(T\right)}B_{\mathrm{tot}}\left(r,t,T\right)=0$$

where $\lambda_{L}\left(T\right)$ is the penetration depth at temperature T [40,41,43] to obtain the magnetization of the superconducting specimen, $M_{\mathrm{AC}}\left(r,\;t,T\right)$. It is easy to understand that $B_{\mathrm{tot}}\left(r,t,T\right)$ has the separation-of-variables form, $B_{\mathrm{tot}}\left(r,t,T\right)=B_{\mathrm{tot}}\left(\rho ,T\right)\cos\left(\omega t\right)\hat{z}$, where the dependence on temperature, T, appears due to the presence of the penetration depth, $\lambda_{L}\left(T\right)$, in the above differential equation. We recall that $\lambda_{L}\left(T\right)$ is a crucial intrinsic parameter that carries information on the mechanism of superconductivity [43]. A basic expression that is employed in most cases relies on the so-called two-fluid model [41,43]:

(16) $$\lambda_{L}\left(T\right)=\sqrt{\frac{m_{\mathrm{sc}}}{\mu_{0}n_{\mathrm{sc}}\left(T\right)q_{\mathrm{sc}}^{2}}}$$

where $m_{\mathrm{sc}}$ and $q_{\mathrm{sc}}^{\;}$ are the mass and the charge of the Cooper pairs (superconducting carriers), respectively, while $n_{\mathrm{sc}}\left(T\right)$ is their density [41,43]. The latter is very important since it exhibits strong dependence on temperature, T. Accordingly, the penetration depth is given by the relation [41,43]

(17) $$\lambda_{L}\left(T\right)=\frac{\lambda_{L}\left(0\right)}{\sqrt{1-{\left(T/T_{c}\right)}^{4}}}$$

From this expression, we see that at T=0 K, $\lambda_{L}\left(T\right)$ obtains its lowest value, $\lambda_{L}\left(0\right)$, while at $T=T_{c}$, $\lambda_{L}\left(T\right)\to \infty$. We stress that for high-T_c_ cuprates such as YBa_2_Cu_3_O_7−δ_ and Bi_2−x_Pb_x_Sr_2_Ca_2_Cu_3_O_10+y_ studied here, the value of the penetration depth at T=0 K, $\lambda_{L}\left(0\right)$, is in the range of a few hundred nanometers. We see that $\lambda_{L}\left(0\right)$ is negligible when compared to the dimensions of meso/macro-scopic specimens so that at T=0 K, $B_{\mathrm{tot}}\left(r,t,T\right)$ penetrates a superconductor only at a surface layer of negligible thickness (~$\lambda_{L}\left(0\right)$). On the other hand, $B_{\mathrm{tot}}\left(r,t,T\right)$ completely penetrates the interior of a superconductor as T approaches $T_{c}$, since $\lambda_{L}\left(T\right)$ diverges.

Returning back to the London differential equation, by introducing the specific form, $B_{\mathrm{tot}}\left(r,t,T\right)=B_{\mathrm{tot}}\left(\rho ,T\right)\cos\left(\omega t\right)\hat{z}$, of the magnetic field, we obtain

(18) $$\frac{d^{2}B_{\mathrm{tot}}\left(\rho ,T\right)}{{d\rho }^{2}}+\frac{1}{\rho }\frac{{\mathrm{dB}}_{\mathrm{tot}}\left(\rho ,T\right)}{{d\rho }^{\;}}-\frac{1}{\lambda_{L}^{2}\left(T\right)}B_{\mathrm{tot}}\left(\rho ,T\right)=0$$

This is a kind of modified Bessel differential equation [44] that has the following solution:

(19) $$B_{\mathrm{tot}}\left(\rho ,T\right)=C_{1}I_{0}\left(\frac{\rho }{\lambda_{L}\left(T\right)}\right)+C_{2}K_{0}\left(\frac{\rho }{\lambda_{L}\left(T\right)}\right)$$

Here, the so-called modified Bessel functions of order zero are introduced, of first, ${Ι}_{0}\left(x\right)$, and second, $K_{0}\left(x\right)$, kinds. The constants $C_{1}$ and $C_{2}$ are found by means of the boundary conditions. The first refers to the z-axis (ρ=0), while the second relates to the surface of the cylinder (ρ=a) [28,41,45,46]. It comes out that

(20) $$B_{\mathrm{tot}}\left(\rho ,t,T\right)=B_{0}\frac{I_{0}\left(\frac{\rho }{\lambda_{L}\left(T\right)}\right)}{I_{0}\left(\frac{a}{\lambda_{L}\left(T\right)}\right)}\cos\left(\omega t\right)\hat{z}$$

where $B_{0}=\mu_{0}H_{0}$. Next, by using the basic relation, $B_{\mathrm{tot}}\left(r,t,T\right)=\mu_{0}\left(H_{\mathrm{ext}}\left(r,t\right)+M_{\mathrm{AC}}\left(r,t,T\right)\right)$, we immediately obtain the magnetization, $M_{\mathrm{AC}}\left(r,t,T\right)$, of the superconducting cylinder as follows:

(21) $$M_{\mathrm{AC}}\left(r,t,T\right)=-\left(1-\frac{I_{0}\left(\frac{\rho }{\lambda_{L}\left(T\right)}\right)}{I_{0}\left(\frac{a}{\lambda_{L}\left(T\right)}\right)}\right)H_{0}\cos\left(\omega t\right)\hat{z}$$

else

(22) $$M_{\mathrm{AC}}\left(r,t,T\right)=-\left(1-\frac{I_{0}\left(\frac{\rho }{\lambda_{L}\left(T\right)}\right)}{I_{0}\left(\frac{a}{\lambda_{L}\left(T\right)}\right)}\right)H_{\mathrm{ext}}\left(r,t\right)$$

As expected, $M_{\mathrm{AC}}\left(r,t,T\right)$ is *linear* in respect to the external magnetic field, $H_{\mathrm{ext}}\left(r,t\right)$, so that we easily recover the *linear* ACMS of the superconducting cylinder, given by the relation

(23) $$\chi_{m,\mathrm{AC}}\left(r,T\right)=-\left(1-\frac{I_{0}\left(\frac{\rho }{\lambda \left(T\right)}\right)}{I_{0}\left(\frac{a}{\lambda \left(T\right)}\right)}\right)$$

Once we have obtained $M_{\mathrm{AC}}\left(r,t,T\right)$ through Relations (21) and (22), we are able to find $m_{\mathrm{AC},\mathrm{SC}}\left(t,T\right)$ through Relation (3). Subsequently, ${\mathrm{dm}}_{\mathrm{AC}}\left(t,T\right)/\mathrm{dt}$ is easily recovered so that the real and imaginary DC voltage signals, $V_{\mathrm{DC}}^{/}\left(T\right)$ and $V_{\mathrm{DC}}^{//}\left(T\right)$, given by the LIA at its outputs, can be ultimately obtained through Relations (11) and (13) and (12) and (14), respectively. Following this procedure, after relatively simple algebraic calculations, by using the property of the modified Bessel functions, $\int I_{0}\left(x\right)\mathrm{xdx}={\mathrm{xI}}_{1}\left(x\right)$ [44], it follows that

(24) $$\frac{{\mathrm{dm}}_{\mathrm{AC}}\left(t,T\right)}{\mathrm{dt}}=V_{\mathrm{SC}}\left(1-2\frac{\lambda \left(T\right)}{a}\frac{I_{1}\left(\frac{a}{\lambda \left(T\right)}\right)}{I_{0}\left(\frac{a}{\lambda \left(T\right)}\right)}\right){\omega H}_{0}\sin\left(\omega t\right)$$

else

(25) $$\frac{{\mathrm{dm}}_{\mathrm{AC}}\left(t,T\right)}{\mathrm{dt}}={\left(\frac{{\mathrm{dm}}_{\mathrm{AC}}\left(t,T\right)}{\mathrm{dt}}\right)}_{0}\sin\left(\omega t\right)$$

where the desired amplitude of the time derivative of the magnetic dipole moment is given by

(26) $${\left(\frac{{\mathrm{dm}}_{\mathrm{AC}}\left(t,T\right)}{\mathrm{dt}}\right)}_{0}=V_{\mathrm{SC}}\left(1-2\frac{\lambda \left(T\right)}{a}\frac{I_{1}\left(\frac{a}{\lambda \left(T\right)}\right)}{I_{0}\left(\frac{a}{\lambda \left(T\right)}\right)}\right){\omega H}_{0}$$

In the above expressions, $I_{1}\left(x\right)$ is the modified Bessel function of the first kind of first order and $V_{\mathrm{SC}}={\pi a}^{2}d$ is the volume of the superconducting cylinder corresponding to a finite length, d. Of particular importance is the term in parentheses on the right side of Relations (24) and (26), which is opposite to the spatial mean value, $\langle \chi_{m,\mathrm{AC}}^{\;}\left(T\right)\rangle =\left(1/V_{\mathrm{SC}}\right)\int_{V_{\mathrm{SC}}}^{\;}\chi_{m,\mathrm{AC}}\left(r,T\right)\mathrm{dV}$, of the linear ACMS function, $\chi_{m,\mathrm{AC}}\left(r,T\right)$. Indeed, by using Relation (23) and the property of the Bessel functions $\int I_{0}\left(x\right)\mathrm{xdx}={\mathrm{xI}}_{1}\left(x\right)$ [44], after relatively simple algebraic calculations, it follows that

(27) $$\langle \chi_{m,\mathrm{AC}}^{\;}\left(T\right)\rangle =\frac{1}{V_{\mathrm{SC}}}\int_{V_{\mathrm{SC}}}^{\;}-\left(1-\frac{I_{0}\left(\frac{\rho }{\lambda \left(T\right)}\right)}{I_{0}\left(\frac{a}{\lambda \left(T\right)}\right)}\right)\mathrm{dV}=-\left(1-2\frac{\lambda \left(T\right)}{a}\frac{I_{1}\left(\frac{a}{\lambda \left(T\right)}\right)}{I_{0}\left(\frac{a}{\lambda \left(T\right)}\right)}\right)$$

Therefore, by using Relations (26) and (27), we see that the desired amplitude of the alternating magnetic dipole moment of the specimen is given by

(28) $${\left(\frac{{\mathrm{dm}}_{\mathrm{AC}}\left(t,T\right)}{\mathrm{dt}}\right)}_{0}=V_{\mathrm{SC}}\left(-\langle \chi_{m,\mathrm{AC}}\left(T\right)\rangle \right){\omega H}_{0}\;$$

It is worth emphasizing that the above amplitude is well defined, ${\left({\mathrm{dm}}_{\mathrm{AC}}\left(t,T\right)/\mathrm{dt}\right)}_{0}\geq 0$, as can be demonstrated by the behavior of the modified Bessel functions, $I_{0}\left(x\right)$ and $I_{1}\left(x\right)$, in the limiting cases of small and large arguments [44]. Specifically, for small arguments, $I_{0}\left(x\right)\approx 1$ and $I_{1}\left(x\right)\approx x/2$, while for large arguments, $I_{0}\left(x\right)=I_{1}\left(x\right)\approx \exp\left(x⁄\sqrt{2\pi x}\;\right)$, where $x=a/\lambda \left(T\right)$. Thus, for small arguments ($a\ll \lambda \left(T\right)$; i.e., $T\to T_{c}$), we obtain $\langle \chi_{m,\mathrm{AC}}^{\;}\left(T\right)\rangle =0$, while for large arguments ($a\gg \lambda \left(T\right)$; i.e., T→0), we obtain $\langle \chi_{m,\mathrm{AC}}^{\;}\left(T\right)\rangle =-1$. Consequently, in the superconducting state, $0\leq T\leq T_{c}$, we have $-1\leq \;\langle \chi_{m,\mathrm{AC}}^{\;}\left(T\right)\rangle \;\leq 0$, as expected [40,41,42].

Substituting Relation (28) into Relations (13) and (14), for the real and imaginary parts of the DC voltage signals provided by the LIA at its respective outputs, we obtain

(29) $$V_{\mathrm{DC}}^{/}\left(T\right)=-{Β}_{0}{\omega V}_{\mathrm{SC}}\left(-\langle \chi_{m,\mathrm{AC}}\left(T\right)\rangle \right)\times F_{\mathrm{PUC}-\mathrm{SDC}}\times F_{\mathrm{LIA}}^{/}$$

and

(30) $$V_{\mathrm{DC}}^{//}\left(T\right)=-{Β}_{0}{\omega V}_{\mathrm{SC}}\left(-\langle \chi_{m,\mathrm{AC}}\left(T\right)\rangle \right)\times F_{\mathrm{PUC}-\mathrm{SDC}}\times F_{\mathrm{LIA}}^{//}$$

where ${Β}_{0}=\mu_{0}H_{0}$. Going a step further, we define the Excitation Function, $F_{\mathrm{EF}}$, of the external trigger (i.e., the external magnetic field in this case) applied to the physical system, and the Response Function $F_{\mathrm{RF}}$ of the physical system (i.e., the superconducting cylinder in this case) to the excitation, through the following relations:

(31) $$F_{\mathrm{EF}}={Β}_{0}\omega =\sqrt{2}B_{\mathrm{AC}}^{\mathrm{rms}}\omega$$

(32) $$F_{\mathrm{RF}}=V_{\mathrm{SC}}\left(-\langle \chi_{m,\mathrm{AC}}\left(T\right)\rangle \right)$$

where $V_{\mathrm{SC}}={\pi a}^{2}d$ is the volume of the superconductor corresponding to a certain length, d. Adopting these definitions, Relations (29) and (30) become the following:

(33) $$V_{\mathrm{DC}}^{/}\left(T\right)=-F_{\mathrm{EF}}\times F_{\mathrm{RF}}\times F_{\mathrm{PUC}-\mathrm{SDC}}\times F_{\mathrm{LIA}}^{/}$$

and

(34) $$V_{\mathrm{DC}}^{//}\left(T\right)=-F_{\mathrm{EF}}\times F_{\mathrm{RF}}\times F_{\mathrm{PUC}-\mathrm{SDC}}\times F_{\mathrm{LIA}}^{//}$$

Here, let us discuss these final relations in detail to clarify their validity, investigate the range of their applicability and ultimately document their importance. First, we see that these DC voltage output signals, $V_{\mathrm{DC}}^{/}\left(T\right)$ and $V_{\mathrm{DC}}^{//}\left(T\right)$, follow a separation-of-variables scheme; they are the product of four different functions, the Excitation ($F_{\mathrm{EF}}$; Relation (31)), the Response ($F_{\mathrm{RF}}$; Relation (32)), the Sensing ($F_{\mathrm{PUC}-\mathrm{SDC}}$; Relation (8)) and the two Transfer ones ($F_{\mathrm{LIA}}^{/}$ and $F_{\mathrm{LIA}}^{//}$; Relations (9) and (10), respectively). In these expressions of $V_{\mathrm{DC}}^{/}\left(T\right)$ and $V_{\mathrm{DC}}^{//}\left(T\right)$, we explicitly show only their dependence on temperature, T. Obviously, both $V_{\mathrm{DC}}^{/}\left(T\right)$ and $V_{\mathrm{DC}}^{//}\left(T\right)$ depend on a plethora of parameters, such as (i) the angular frequency, ω, and the amplitude, ${Β}_{0}$, of the external AC magnetic field, $B_{\mathrm{ext}}\left(r,t\right)$; (ii) the dimensions of the superconducting cylinder (i.e., radius, a) and the penetration depth, $\lambda_{L}\left(T\right)$; (iii) the characteristics of the four coaxial PUCs in the SDC (i.e., total number of turns, N, inner radius, $R_{1}$, outer radius $R_{2}$, thickness, $D=R_{2}-R_{1}$, length, L, of each identical PUC, as well as their distance); and (iv) the parameters employed in the LIA, i.e., the sensitivity and the relative phase/angle, θ. The latter parameter is very important; thus, it deserves special attention. We underline that, by definition, the first three functions $F_{\mathrm{EF}}$, $F_{\mathrm{RF}}$ and $F_{\mathrm{PUC}-\mathrm{SDC}}$ are always positive. On the contrary, the last ones, $F_{\mathrm{LIA}}^{/}$ in Relation (33) and $F_{\mathrm{LIA}}^{//}$ in Relation (34), depend on the choice of the relative phase/angle θ. Specifically, the relative phase/angle θ of the LIA provides an internal ‘degree-of-freedom’ that appears in both trigonometric coefficients, the cosinusoidal and the sinusoidal, and enables us to selectively adjust the respective output signals, $V_{\mathrm{DC}}^{/}\left(T\right)$ (Relation (33)) and $V_{\mathrm{DC}}^{//}\left(T\right)$ (Relation (34)), on a comparative basis. *This facility of every LIA enables an experienced user to ascribe the proper physical content, both quantitatively and qualitatively, to the output signals,* $V_{DC}^{/}\left(T\right)$ *and* $V_{DC}^{//}\left(T\right)$. For instance, in our case, in the superconducting state, $T<T_{c}$, we should observe a negative real/in-phase/cosinusoidal signal (diamagnetic response), $V_{\mathrm{DC}}^{/}\left(T\right)\leq 0$, and a positive imaginary/out-of-phase/sinusoidal signal (losses should always be positive), $V_{\mathrm{DC}}^{//}\left(T\right)\geq 0$; thus, the overall phase/angle θ should be 3π/2≤θ≤2π.

Until now, we have theoretically treated a superconducting cylinder of infinite length subjected to an external magnetic field parallel to its axis and used this theoretical information in the mathematical modeling of our ACMS experimental set-up. Obviously, this is an ideal case where demagnetizing effects are absent. However, in reality, we use specimens of finite dimensions so that demagnetizing effects are always present. Accordingly, based on our as-recorded ACMS experimental data, we can directly calculate $\langle \chi_{m,\mathrm{AC}}\left(T\right)\rangle$ by using Relations (33) and (34) (accompanied by (8), (9), (10), (31) and (32)). Apparently, $\langle \chi_{m,\mathrm{AC}}\left(T\right)\rangle$ coincides with the so-called *extrinsic* ACMS, $\langle \chi_{m,\mathrm{AC}}^{\mathrm{ext}}\left(T\right)\rangle$, which depends on the shape and dimensions of each particular specimen under investigation. To successfully recover the truly *intrinsic* ACMS of the parent material, $\langle \chi_{m,\mathrm{AC}}^{\mathrm{int}}\left(T\right)\rangle$, from the *extrinsic* ACMS, $\langle \chi_{m,\mathrm{AC}}^{\mathrm{ext}}\left(T\right)\rangle$, we have to take into account demagnetizing effects that are quantified through the so-called demagnetizing factor, N. To this end, we recall computational results from the literature [27,28]. Specifically, for the case of diamagnetic specimens of standard shape, the demagnetizing factor, N, is given by simple, approximate relations. For instance, $N^{-1}=1+1.6\left(c/a\right)$ for a cylinder of diameter 2a and height 2c, and $N^{-1}=1+\left(3/4\right)\left(c/a\right)\left(1+a/b\right)$ for a rectangular parallelepiped of sides 2a and 2b and height 2c [27,28]. Thus, by taking into account the demagnetizing factor, N, we can calculate the spatial mean value of the *intrinsic* ACMS, $\langle \chi_{m,\mathrm{AC}}^{\mathrm{int}}\left(T\right)\rangle$, of the parent material from the spatial mean value of the *extrinsic* ACMS, $\langle \chi_{m,\mathrm{AC}}^{\mathrm{ext}}\left(T\right)\rangle$, of each specimen, recorded in our ACMS measurements, from the relation [27,28]

(35) $$\langle \chi_{m,\mathrm{AC}}^{\mathrm{int}}\left(T\right)\rangle =\frac{\langle \chi_{m,\mathrm{AC}}^{\mathrm{ext}}\left(T\right)\rangle }{1-N\langle \chi_{m,\mathrm{AC}}^{\mathrm{ext}}\left(T\right)\rangle }$$

## 5. Experimental Results

To test the detailed model of the ACMS experimental set-up presented above for the case of a superconducting cylinder, we performed detailed measurements on poly-crystalline, bulk specimens of high-T_c_ superconductors YBa_2_Cu_3_O_7−δ_ and Bi_2−x_Pb_x_Sr_2_Ca_2_Cu_3_O_10+y_, shaped in cylinder form. Below, the finite length of the superconducting cylinder is taken into account by means of Relation (35). This is the only important approximation employed in our analysis. As shown below, the comparison of the mathematical modeling with the experimental data evidences that the introduced demagnetizing factor captures the underlying processes quite effectively. Finally, to overcome the porosity and the non-linear behavior of the aforementioned poly-crystalline samples, we investigated a single crystal of YBa_2_Cu_3_O_7−δ_ in the form of a thin plate.

### 5.1. Poly-Crystalline Cylindrical Specimen of High-T_c_ YBa_2_Cu_3_O_7−δ_

**Figure 3:**
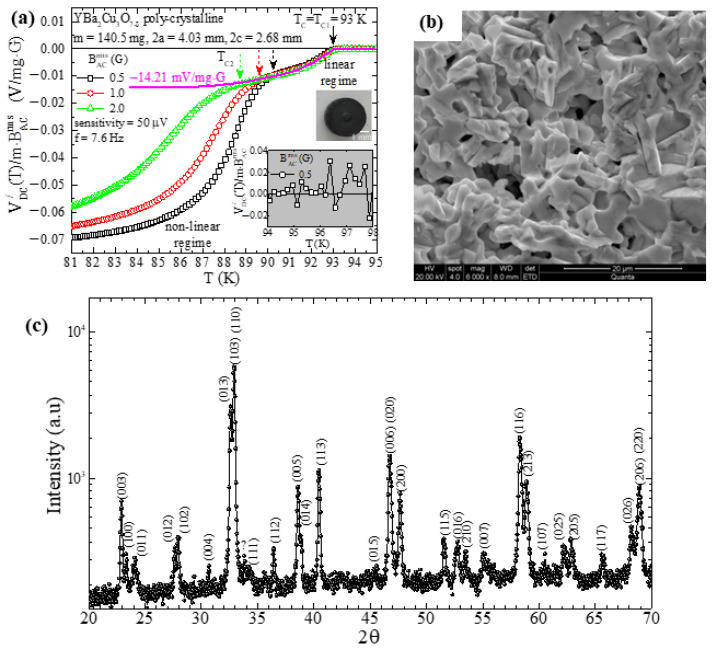
Data of poly-crystalline, cylinder-shaped specimen of YBa_2_Cu_3_O_7−δ_. (**a**) Plot of the variation in temperature (T) of the real part DC voltage signal, $V_{\mathrm{DC}}^{/}\left(T\right)$, reduced to the mass (m) of the specimen and to the rms value of the externally applied magnetic field ($B_{\mathrm{AC}}^{\mathrm{rms}}$) for three different measurements ($B_{\mathrm{AC}}^{\mathrm{rms}}=0.5,\;1.0$ and 2.0 G). Upper inset: the specimen has mass m = 140.5 mg, diameter 2a = 4.03 mm and height 2c = 2.68 mm. Lower inset: normal-state signal in the temperature range 94 K≤T≤98 K, to obtain the mean value ($\langle S\rangle$) and the standard deviation (SD) of $V_{\mathrm{DC}}^{/}\left(T\right)/\left(m\cdot B_{\mathrm{AC}}^{\mathrm{rms}}\right)$; $\langle S\rangle \pm \mathrm{SD}=-2.45\pm 15.44\;\left(\mu V/\mathrm{mg}\cdot G\right)$. The magenta, thick curve refers to the theoretically expected behavior of the high-temperature linear part of the experimental data, when extrapolated in the low-temperature regime saturates at $-14.21\;\left(\mathrm{mV}/\mathrm{mg}\cdot G\right)$. (**b**) Representative SEM photograph of the surface of the exact same specimen at magnification 6000×. (**c**) Representative XRD data obtained in a powdered sample of the exact same specimen.

Starting with the poly-crystalline, bulk, high-T_c_ superconductor YBa_2_Cu_3_O_7−δ_, in Figure 3a–c, we show representative data of ACMS, SEM and XRD, respectively. Also, in the upper inset of Figure 3a, we show a photo of the poly-crystalline cylinder specimen (top view), with diameter 4.03 mm, height 2.68 mm and mass 140.5 mg (the sample was subjected to sintering at 920 °C for 24 h). In Figure 3a, we present ACMS data. Specifically, we show the variation in the temperature (T) of the real part DC voltage signal, $V_{\mathrm{DC}}^{/}\left(T\right)$, when reduced to the mass of the specific specimen and to the rms value of the externally applied magnetic field. Three different measurements are presented, where $B_{\mathrm{AC}}^{\mathrm{rms}}=0.5,\;1.0$ and 2.0 G. The presentation of the reduced signal, $V_{\mathrm{DC}}^{/}\left(T\right)/\left(m\cdot B_{\mathrm{AC}}^{\mathrm{rms}}\right)$ (instead of $V_{\mathrm{DC}}^{/}\left(T\right)$), is very convenient because it enables the direct quantitative comparison of measurements obtained at specimens of different mass, for different rms values of the external magnetic field. On the other hand, in these three measurements, the frequency was the same, $f_{\mathrm{AC}}=7.6\;\mathrm{Hz}$, and the sensitivity was also the same, sensitivity=50 μV.

In these data, we clearly see that the transition from the normal to the superconducting state evolves into two distinct stages. The first transition is observed at $T_{c}=T_{c1}=93\;K$ and does not depend on $B_{\mathrm{AC}}^{\mathrm{rms}}$, while the second one occurs at a much lower temperature, $T_{c2}$, which strongly depends on $B_{\mathrm{AC}}^{\mathrm{rms}}$, thus, $T_{c2}\left(B_{\mathrm{AC}}^{\mathrm{rms}}\right)$. Interestingly, the temperature range between the fixed $T_{c1}$ and $T_{c2}\left(B_{\mathrm{AC}}^{\mathrm{rms}}\to 0\right)$ is governed by a linear response on $B_{\mathrm{AC}}^{\mathrm{rms}}$ (i.e., $V_{\mathrm{DC}}^{/}\left(T\right)/\left(m\cdot B_{\mathrm{AC}}^{\mathrm{rms}}\right)$ does not depend on $B_{\mathrm{AC}}^{\mathrm{rms}}$). On the contrary, for temperatures $T\leq T_{c2}\left(B_{\mathrm{AC}}^{\mathrm{rms}}\to 0\right)$, the DC voltage signal, $V_{\mathrm{DC}}^{/}\left(T\right)$, exhibits a strongly non-linear response on $B_{\mathrm{AC}}^{\mathrm{rms}}$ as evidenced by the behavior of the reduced signal $V_{\mathrm{DC}}^{/}\left(T\right)/\left(m\cdot B_{\mathrm{AC}}^{\mathrm{rms}}\right)$ as well. The underlying mechanism responsible for this behavior originates from the inter-grain and intra-grain establishment of superconductivity as temperature is progressively lowered [47,48,49,50]. This issue is beyond the scope of the present work and will be discussed elsewhere. In the lower inset, we focus on the data of the normal-state temperature range, 94 K≤T≤98 K, obtained for $B_{\mathrm{AC}}^{\mathrm{rms}}=0.5\;G$. There, it is expected that the mean value of the signal (<S>) should be zero so that a reliable estimation of the noise level can be performed from the standard deviation (SD). The statistics of these data evidenced that $\langle S\rangle \pm \mathrm{SD}=-2.45\pm 15.44\;\left(\mu V/\mathrm{mg}\cdot G\right)$, so that the signal-to-noise ratio reaches $\left|\mathrm{signal}/\mathrm{noise}\right|=\left(0.07/15.44\right)10^{6}\approx 4.500$, an extremely high value.

Figure 3b shows a representative SEM photograph, obtained at magnification 6000×, from the surface of the exact same specimen. We see that the specimen exhibits a noticeable porosity, with grains/crystallites of dimensions up to tens of micrometers. Porosity imprints limitations in the desired theoretical modeling of the Response Function, $F_{\mathrm{RF}}$ (Relation (32)), of the superconducting specimen to the external magnetic field. In the simple case, porosity reduces the so-called superconducting volume fraction, a fact that we take into consideration below. Also, the poly-crystalline specimen shown in Figure 3b is actually a three-dimensional network of superconducting grains connected with ‘weak links’ [51,52,53,54,55]. As evidenced in the ACMS data of Figure 3a, this complex superconducting network exhibits a mixture of both linear and highly non-linear responses to the externally applied magnetic field that is quite difficult to model by theory. Nevertheless, below, we compare the experimental data with the theoretically expected ones by using Relation (33). The thick magenta curve refers to the expected behavior of the high-temperature, linear part of the experimental data when extrapolated in the low-temperature regime saturates at $-14.21\;\left(\mathrm{mV}/\mathrm{mg}\cdot G\right)$.

Here, let us describe in detail how the theoretically expected data are obtained by using Relation (33) for the case where $B_{\mathrm{AC}}^{\mathrm{rms}}=0.5\;G$. To this end, we need to estimate the relevant functions $F_{\mathrm{EF}}$, $F_{\mathrm{RF}}$, $F_{\mathrm{PUC}-\mathrm{SDC}}$ and $F_{\mathrm{LIA}}^{/}$, one by one. The Excitation Function (Relation (31)) is $F_{\mathrm{EF}}={Β}_{0}\omega =\left(0.5\sqrt{2}\cdot 10^{-4}\;T\right)\left(47.8\;\mathrm{Hz}\right)=3.38\cdot 10^{-3}\;\left(T\cdot \mathrm{Hz}\right)$. The Response Function (Relation (32)) reads $F_{\mathrm{RF}}=V_{\mathrm{SC}}\left(-\langle \chi_{m,\mathrm{AC}}\left(T\right)\rangle \right)=\left(3.42\cdot 10^{-8}{\;m}^{3}\right)\left(1.94\right)=6.63\cdot 10^{-8}{\;m}^{3}$, where $\langle \chi_{m,\mathrm{AC}}\left(T\right)\rangle =\langle \chi_{m,\mathrm{AC}}^{\mathrm{ext}}\left(T\right)\rangle =\langle \chi_{m,\mathrm{AC}}^{\mathrm{int}}\left(T\right)\rangle /\left(1-N\langle \chi_{m,\mathrm{AC}}^{\mathrm{int}}\left(T\right)\rangle \right)=-1.94$ is the *extrinsic* ACMS. For its calculation, we use the fact that the *intrinsic* ACMS is $\langle \chi_{m,\mathrm{AC}}^{\mathrm{int}}\left(T\right)\rangle =-1$ (Meissner state) and the demagnetizing factor, N, is given by $N^{-1}=1+1.6c/a=1/0.48$, where 2a = 4.03 mm is the diameter and 2c = 2.68 mm is the height of the superconductor [27,28]. The Sensing Function (Relation (8)), for the following parameters N=675, $R_{1}=2.35\;\mathrm{mm}$, $R_{2}=4.10\;\mathrm{mm}$, $D=R_{2}-R_{1}=1.75\;\mathrm{mm}$ and L=10.78 mm (total number of turns, inner radius, outer radius, thickness and length of each identical PUC, respectively), results in $F_{\mathrm{PUC}-\mathrm{SDC}}=\left(N/D\right)\ln\left(\left(R_{2}+\sqrt{R_{2}^{2}+L^{2}}\right)/\left(R_{1}+\sqrt{R_{1}^{2}+L^{2}}\right)\right)=10,7411{\;m}^{-1}$. Finally, the real part Transfer Function of the LIA (Relation (9)), for sensitivity = 50 μV and cosθ = 1, gives $F_{\mathrm{LIA}}^{/}=(1/\sqrt{2})(10/\mathrm{sensitivity})=14,1421\;V$.

Before we proceed with the final estimation of the theoretically expected data, we note that we still have to consider two issues. First, part of the specimen’s volume *is not superconducting at all* due to its inherent porosity (see SEM image in Figure 3b). Second, part of the specimen’s volume *is not superconducting linearly* due to the barrier that appears in the electrical conductivity between grains (see the low-temperature regime of the ACMS experimental data in Figure 3a). By recalling that our investigation refers to the *linear* response of a superconducting specimen, we understand that we have to introduce two correction factors in the nominal volume of our specimen. Thus, the *linearly superconducting*, corrected volume of our specimen should be $V_{\mathrm{SC}-\mathrm{cor}}=C_{p}C_{\mathrm{lr}}V_{\mathrm{SC}}$, where $C_{p}\leq 1$ is the correction factor due to the porosity (reduced superconducting volume fraction) and $C_{\mathrm{lr}}\leq 1$ is the correction factor due to the existence of a non-linear response (reduced linearly responding superconducting volume fraction). Accordingly, the Response Function should be $F_{\mathrm{RF}}=V_{\mathrm{SC}-\mathrm{cor}}\left(-\langle \chi_{m,\mathrm{AC}}\left(T\right)\rangle \right)$. Based on our detailed SEM data, we estimate that $0.75\leq C_{p}\leq 0.95$, while based on our ACMS data, we estimate that $0.2\leq C_{\mathrm{lr}}\leq 0.4$, where we assume that any proportionality between the linear and non-linear parts of the signal directly translates to the respective linearly and non-linearly responding volume fractions of the specimen. Specifically, from the data presented in Figure 3a that refer to the case where $B_{\mathrm{AC}}^{\mathrm{rms}}=0.5\;G$, we have $C_{\mathrm{lr}}=\left(-14.21\right)/\left(-70.00-\left(-14.21\right)\right)=0.26$. Finally, once $0.75\leq C_{p}\leq 0.95$, the Response Function ranges within $1.27\cdot 10^{-8}{\;m}^{3}\leq F_{\mathrm{RF}}\leq 1.60\cdot 10^{-8}{\;m}^{3}$. Eventually, by recalling Relation (33), the theoretically expected signal should range within $-0.82\;V\leq V_{\mathrm{DC}-\mathrm{the}}^{/}\leq -0.65\;V$, or else, the respective reduced signal should be $-11.72\;\mathrm{mV}/\mathrm{mg}\cdot G\leq V_{\mathrm{DC}-\mathrm{the}}^{/}/\left(m\cdot B_{\mathrm{AC}}^{\mathrm{rms}}\right)\leq -9.25\;\mathrm{mV}/\mathrm{mg}\cdot G$. The comparison with the experimental data, $V_{\mathrm{DC}-\exp}^{/}$, evidences that the percentage difference ranges within

(36) $$17.5\%\leq \frac{V_{\mathrm{DC}-\mathrm{the}}^{/}-V_{\mathrm{DC}-\exp}^{/}}{V_{\mathrm{DC}-\exp}^{/}}100\%\leq 34.9\%$$

Thus, for the case of YBa_2_Cu_3_O_7−δ_, the agreement between the purely experimental and the theoretically expected results is quite reasonable, given that in our mathematical model, we did not employ any crude assumption/approximation.

Finally, Figure 3c shows representative XRD data obtained in a powdered sample of the exact same specimen, where all peaks are assigned to YBa_2_Cu_3_O_7−δ_ with δ ≈ 0.08, as expected from the optimum T_c_ evidenced in Figure 3a [30,32].

### 5.2. Poly-Crystalline Cylindrical Specimen of High-T_c_ Bi_2−x_Pb_x_Sr_2_Ca_2_Cu_3_O_10+y_

**Figure 4:**
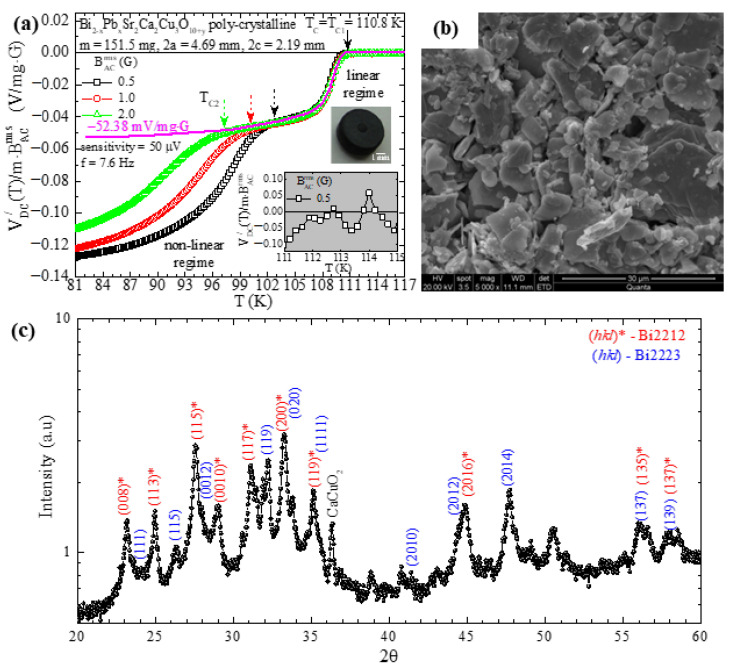
Data of poly-crystalline, cylinder-shaped specimen of Bi_2−x_Pb_x_Sr_2_Ca_2_Cu_3_O_10+y_. (**a**) Plot of the variation in temperature (T) of the real part DC voltage signal, $V_{\mathrm{DC}}^{/}\left(T\right)$, reduced to the mass (m) of the specimen and to the rms value of the externally applied magnetic field ($B_{\mathrm{AC}}^{\mathrm{rms}}$) for three different measurements ($B_{\mathrm{AC}}^{\mathrm{rms}}=0.5,\;1.0$ and 2.0 G). Upper inset: the specimen has mass m = 151.5 mg, diameter 2a = 4.69 mm and height 2c = 2.19 mm. Lower inset: normal-state signal in the temperature range 111 K≤T≤115 K, to obtain the mean value ($\langle S\rangle$) and the standard deviation (SD) of $V_{\mathrm{DC}}^{/}\left(T\right)/\left(m\cdot B_{\mathrm{AC}}^{\mathrm{rms}}\right)$; $\langle S\rangle \pm \mathrm{SD}=-16.93\pm 27.16\;\left(\mu V/\mathrm{mg}\cdot G\right)$. The thick magenta curve refers to the theoretically expected behavior of the high-temperature linear part of the experimental data, when extrapolated in the low-temperature regime saturates at $-52.38\;\left(\mathrm{mV}/\mathrm{mg}\cdot G\right)$. (**b**) Representative SEM photograph from the surface of the exact same specimen at magnification 5000×. (**c**) Representative XRD data obtained in a powdered sample of the exact same specimen.

We proceed with the poly-crystalline, bulk, high-T_c_ superconductor Bi_2−x_Pb_x_Sr_2_Ca_2_Cu_3_O_10+y_. Figure 4a–c show representative data of ACMS, SEM and XRD, respectively. Also, in the upper inset of Figure 4a, we show a photo of the poly-crystalline cylinder specimen (perspective view), with diameter 4.69 mm, height 2.19 mm and mass 151.5 mg (the sample was subjected to sintering at 845 °C for 24 h). In Figure 4a, we present the respective ACMS data for Bi_2−x_Pb_x_Sr_2_Ca_2_Cu_3_O_10+y_, as shown above for YBa_2_Cu_3_O_7−δ_, obtained at the exact same parameters (rms value, $B_{\mathrm{AC}}^{\mathrm{rms}}=0.5,\;1.0$ and 2.0 G, and frequency, $f_{\mathrm{AC}}=7.6\;\mathrm{Hz}$, of the externally applied magnetic field, and sensitivity of the LIA, sensitivity=50 μV).

Again, we clearly see that the transition from the normal to the superconducting state evolves into two distinct stages. The first transition is observed at $T_{c}=T_{c1}=110.8\;K$ and does not depend on $B_{\mathrm{AC}}^{\mathrm{rms}}$, while the second one occurs at $T_{c2}$, which strongly depends on $B_{\mathrm{AC}}^{\mathrm{rms}}$, thus, $T_{c2}\left(B_{\mathrm{AC}}^{\mathrm{rms}}\right)$. In the temperature range from $T_{c1}=110.8\;K$ to $T_{c2}\left(B_{\mathrm{AC}}^{\mathrm{rms}}\to 0\right)=101.7\;K$, the response of the specimen is linear on $B_{\mathrm{AC}}^{\mathrm{rms}}$, since the reduced signal, $V_{\mathrm{DC}}^{/}\left(T\right)/(m\cdot B_{\mathrm{AC}}^{\mathrm{rms}})$, does not depend on $B_{\mathrm{AC}}^{\mathrm{rms}}$. On the contrary, for temperatures $T\leq T_{c2}\left(B_{\mathrm{AC}}^{\mathrm{rms}}\to 0\right)=101.7\;K$, the recorded real part DC voltage signal, $V_{\mathrm{DC}}^{/}\left(T\right)$, exhibits a strongly non-linear response on $B_{\mathrm{AC}}^{\mathrm{rms}}$ as clearly evidenced by the behavior of the reduced signal, $V_{\mathrm{DC}}^{/}\left(T\right)/\left(m\cdot B_{\mathrm{AC}}^{\mathrm{rms}}\right)$. As discussed above for YBa_2_Cu_3_O_7−δ_, the underlying mechanism responsible for this behavior originates from the inter-grain and intra-grain establishment of superconductivity as temperature is progressively lowered [47,48,49,50]. This issue is beyond the scope of the present work and will be discussed elsewhere. In the lower inset, we focus on the data of the normal-state temperature range, 111 K≤T≤115 K, obtained for $B_{\mathrm{AC}}^{\mathrm{rms}}=0.5\;G$, to estimate the noise level from the standard deviation (SD) of the signal. The statistics of these data evidenced that $\langle S\rangle \pm \mathrm{SD}=-16.93\pm 27.16\;\left(\mu V/\mathrm{mg}\cdot G\right)$, so that the signal-to-noise ratio reaches $\left|\mathrm{signal}/\mathrm{noise}\right|=\left(0.128/27.16\right)10^{6}\approx 4.700$, which is in the same order of magnitude as that reported above for YBa_2_Cu_3_O_7−δ_.

Figure 4b shows a representative SEM photograph, obtained at magnification 5000×, from the surface of the exact same specimen. We see that the specimen exhibits a noticeable porosity, with grains/crystallites of dimensions up to tens of micrometers. As discussed above, the porosity and the three-dimensional network of superconducting grains connected with ‘weak links’ [51,52,53,54,55] introduce strong limitations in the desired theoretical modeling of the Response Function, $F_{\mathrm{RF}}$ (Relation (32)), of the superconducting specimen to the external magnetic field. Nevertheless, as carried out above for the case of YBa_2_Cu_3_O_7−δ_, here, we will also try to model the experimental data with the theoretically expected ones by using Relation (33). The thick magenta curve refers to the expected behavior of the high-temperature, linear part of the experimental data, when extrapolated in the low-temperature regime saturates at $-52.38\;\left(\mathrm{mV}/\mathrm{mg}\cdot G\right)$.

Following the procedure of Section 5.1, here, we describe in brief how the theoretically expected data are obtained by using Relation (33) for the case of poly-crystalline Bi_2−x_Pb_x_Sr_2_Ca_2_Cu_3_O_10+y_. Since the excitation field, the geometrical characteristics of the PUCs, and the sensitivity of the LIA are the same as in Section 5.1, the respective functions, $F_{\mathrm{EF}}$, $F_{\mathrm{PUC}-\mathrm{SDC}}$ and $F_{\mathrm{LIA}}^{/}$, are exactly the same: $F_{\mathrm{EF}}=3.38\cdot 10^{-3}\;\left(T\cdot \mathrm{Hz}\right)$, $F_{\mathrm{PUC}-\mathrm{SDC}}=107411{\;m}^{-1}$ and $F_{\mathrm{LIA}}^{/}=141421\;V$. The only difference is on the Response Function (Relation (32)). Here, we have $F_{\mathrm{RF}}=V_{\mathrm{SC}}\left(-\langle \chi_{m,\mathrm{AC}}\left(T\right)\rangle \right)=\left(3.78\cdot 10^{-8}{\;m}^{3}\right)\;\left(2.34\right)=8.85\cdot 10^{-8}{\;m}^{3}$, where $\langle \chi_{m,\mathrm{AC}}\left(T\right)\rangle =\langle \chi_{m,\mathrm{AC}}^{\mathrm{ext}}\left(T\right)\rangle =\langle \chi_{m,\mathrm{AC}}^{\mathrm{int}}\left(T\right)\rangle /\left(1-N\langle \chi_{m,\mathrm{AC}}^{\mathrm{int}}\left(T\right)\rangle \right)=-2.34$ is the *extrinsic* ACMS. For its calculation, we used the fact that the *intrinsic* ACMS is $\langle \chi_{m,\mathrm{AC}}^{\mathrm{int}}\left(T\right)\rangle =-1$ (Meissner state) and the demagnetizing factor, N, is given by $N^{-1}=1+1.6c/a=1/0.57$, where 2a = 4.69 mm is the diameter and 2c = 2.19 mm is the height of the superconductor [27,28].

As in Section 5.1, the *linearly superconducting*, corrected volume of our specimen should be $V_{\mathrm{SC}-\mathrm{cor}}=C_{p}C_{\mathrm{lr}}V_{\mathrm{SC}}$, where $C_{p}\leq 1$ is the correction factor due to the porosity that reduces the superconducting volume fraction and $C_{\mathrm{lr}}\leq 1$ is the correction factor that takes into consideration the linearly responding superconducting volume fraction. Accordingly, the Response Function should be $F_{\mathrm{RF}}=V_{\mathrm{SC}-\mathrm{cor}}\left(-\langle \chi_{m,\mathrm{AC}}\left(T\right)\rangle \right)$. Based on our detailed SEM data, we estimate that $0.75\leq C_{p}\leq 0.95$, while based on our ACMS data, we estimate that $0.6\leq C_{\mathrm{lr}}\leq 0.8$, where we assume that any proportionality between the linear and non-linear parts of the signal directly translates to the respective linearly and non-linearly responding volume fractions of the specimen. Specifically, from the data presented in Figure 4a that refer to the case where $B_{\mathrm{AC}}^{\mathrm{rms}}=0.5\;G$, we have $C_{\mathrm{lr}}=\left(-52.38\right)/\left(-127.57-\left(-52.38\right)\right)=0.69$. Finally, once $0.75\leq C_{p}\leq 0.95$, the Response Function ranges within $4.62\cdot 10^{-8}{\;m}^{3}\leq F_{\mathrm{RF}}\leq 5.86\cdot 10^{-8}{\;m}^{3}$. Eventually, by recalling Relation (33), the theoretically expected signal should be $-3.00\;V\leq V_{\mathrm{DC}-\mathrm{the}}^{/}\leq -2.37\;V$, or else the respective reduced signal should be $-39.64\;\mathrm{mV}/\mathrm{mg}\cdot G\leq V_{\mathrm{DC}-\mathrm{the}}^{/}/\left(m\cdot B_{\mathrm{AC}}^{\mathrm{rms}}\right)\leq -31.29\;\mathrm{mV}/\mathrm{mg}\cdot G$. The comparison with the experimental data, $V_{\mathrm{DC}-\exp}^{/}$, evidences that the percentage difference ranges within

(37) $$24.3\%\leq \frac{V_{\mathrm{DC}-\mathrm{the}}^{/}-V_{\mathrm{DC}-\exp}^{/}}{V_{\mathrm{DC}-\exp}^{/}}100\%\leq 40.2\%$$

Thus, for the case of Bi_2−x_Pb_x_Sr_2_Ca_2_Cu_3_O_10+y_, the agreement between the purely experimental and the theoretically expected results is quite reasonable as well.

The relatively higher percentage difference observed for the case of Bi_2−x_Pb_x_Sr_2_Ca_2_Cu_3_O_10+y_ in comparison to YBa_2_Cu_3_O_7−δ_ can be ascribed to differences in the underlying physics of the ‘weak links’ between the two cases [51,52,53,54,55]. For instance, for the case of YBa_2_Cu_3_O_7−δ_, the scenario of a second superconducting phase is not at play. This is evidenced by detailed XRD data and the subsequent thorough analysis (a representative XRD pattern is shown in Figure 3c). However, for the case of Bi_2−x_Pb_x_Sr_2_Ca_2_Cu_3_O_10+y_, the situation is surely different since in the particular specimen investigated here, a second superconducting phase, (BiPb)-2212, coexists with the desired phase, (BiPb)-2223. This is clearly evidenced by the respective XRD data in Figure 4c. Thus, for the case of Bi_2−x_Pb_x_Sr_2_Ca_2_Cu_3_O_10+y_, the non-linear response that appears in the low-temperature regime (Figure 4a) is probably motivated and/or promoted by the coexistence of the two different superconducting phases, (BiPb)-2223 and (BiPb)-2212.

The discrepancies raised from the porosity and the non-linear response of these poly-crystalline YBa_2_Cu_3_O_7−δ_ and Bi_2−x_Pb_x_Sr_2_Ca_2_Cu_3_O_10+y_ specimens are removed by using a compact single crystal of YBa_2_Cu_3_O_7−δ_ as discussed in the following subsection.

### 5.3. Single-Crystalline Thin Plate of High-T_c_ YBa_2_Cu_3_O_7−δ_

**Figure 5:**
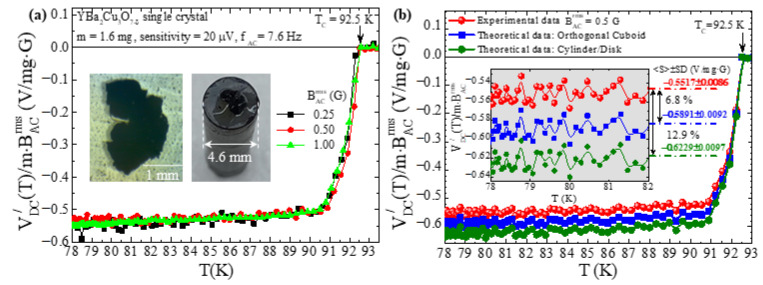
Data of single-crystalline thin-plate YBa_2_Cu_3_O_7−δ_. (**a**) Plot of the variation in temperature (T) of the real part DC voltage signal, $V_{\mathrm{DC}}^{/}\left(T\right)$, when reduced to the mass of the single crystal (m=1.6 mg) and to the rms value of the external magnetic field ($B_{\mathrm{AC}}^{\mathrm{rms}}=0.25,\;0.5\;\mathrm{and}\;1.0\;G$), that is, $V_{\mathrm{DC}}^{/}\left(T\right)/\left(m\cdot B_{\mathrm{AC}}^{\mathrm{rms}}\right)$. Left inset: stereo microscope image of a single crystal of superconductor YBa_2_Cu_3_O_7−δ_ with mass m=1.6 mg (top view). Right inset: the single crystal on the graphite holder (perspective view). (**b**) Plot of experimental data of $V_{\mathrm{DC}}^{/}\left(T\right)/\left(m\cdot B_{\mathrm{AC}}^{\mathrm{rms}}\right)$, obtained for $B_{\mathrm{AC}}^{\mathrm{rms}}=0.5\;G$ (red spheres), together with the theoretically expected ones calculated by means of Relation (33); blue squares and olive circles refer to the case when the single crystal is approximated by an orthogonal cuboid and a cylinder/disc, respectively. In the inset, we focus on the low temperature range, 78 K≤T≤82 K, to obtain the mean value ($\langle S\rangle$) and the standard deviation (SD) of $V_{\mathrm{DC}}^{/}\left(T\right)/\left(m\cdot B_{\mathrm{AC}}^{\mathrm{rms}}\right)$, so that the percentage difference between the experimental and the two theoretical cases is defined (see text for details).

The above discussion evidenced that the porosity and the non-linear response of the poly-crystalline, cylindrical specimens of YBa_2_Cu_3_O_7−δ_ and Bi_2−x_Pb_x_Sr_2_Ca_2_Cu_3_O_10+y_ introduce a high degree of complexity in the theoretical modeling of the Response Function, $F_{\mathrm{RF}}$ (Relation (32)), of the superconducting specimen to the external magnetic field. To overcome this difficulty, here, we focus on compact, single-crystalline YBa_2_Cu_3_O_7−δ_ in the form of a thin plate. This particular single crystal comes from the same batch investigated a long time ago in [31] and is shown in the stereo microscope photo presented in the left inset of Figure 5a. It has an irregular shape (dimensions within 1.0–2.5 mm in the ab plane and thickness about 80 μm in the vertical axis c) and a mass m=1.6 mg. In the stereo microscope photo presented in the right inset of Figure 5b, the single crystal is shown when placed on the graphite holder by means of Apiezon N Grease. Figure 5a shows the variation in temperature (T) of the real part DC voltage signal, $V_{\mathrm{DC}}^{/}\left(T\right)$, when reduced to the mass (m) of the single crystal and to the rms value of the external magnetic field ($B_{\mathrm{AC}}^{\mathrm{rms}}$), that is, $V_{\mathrm{DC}}^{/}\left(T\right)/\left(m\cdot B_{\mathrm{AC}}^{\mathrm{rms}}\right)$. These measurements were obtained at $B_{\mathrm{AC}}^{\mathrm{rms}}=0.25,\;0.5\;\mathrm{and}\;1.0\;G$ and $f_{\mathrm{AC}}=7.6\;\mathrm{Hz}$. Clearly, the single crystal exhibits a linear response on $B_{\mathrm{AC}}^{\mathrm{rms}}$ in all temperature ranges so that a direct comparison with our mathematical model can be performed. Figure 5b focuses on the experimental data obtained for $B_{\mathrm{AC}}^{\mathrm{rms}}=0.5\;G$ (red spheres), together with theoretically expected ones (see below). In the inset of the measurement, we focus on the temperature range 78 K≤T≤82 K of the superconducting state well below the critical temperature, $T_{C}=92.5\;K$, where the measured signal should be constant (the Meissner state of perfect diamagnetism, $\langle \chi_{m,\mathrm{AC}}^{\mathrm{int}}\left(T\right)\rangle =-1$, should have been established). The statistics of the experimental data (red spheres) of the inset show that $\langle S\rangle \pm \mathrm{SD}=-0.5517\pm 0.0086\;\left(V/\mathrm{mg}\cdot G\right)$, so that the signal-to-noise ratio is $\left|\mathrm{signal}/\mathrm{noise}\right|=0.5517/0.0086\approx 64$, a very satisfactory value, considering the extremely small mass of the single crystal (if we obtain the noise level from the data of the normal-state temperature range, 92.5 K≤T≤95.0 K, as was carried out in the case of the poly-crystalline samples discussed above, we obtain $\left|\mathrm{signal}/\mathrm{noise}\right|=0.55170/0.00187\approx 295$).

Figure 5b also presents the theoretically expected data calculated by means of Relation (33) and the procedure of Section 5.1 and Section 5.2, when the single crystal is approximated by an orthogonal cuboid (blue squares) and a cylinder/disc (olive circles). Briefly, the excitation field and the geometrical characteristics of the PUCs are the same as in the poly-crystalline cylindrical specimens of YBa_2_Cu_3_O_7−δ_ and Bi_2−x_Pb_x_Sr_2_Ca_2_Cu_3_O_10+y_ of Section 5.1 and Section 5.2, respectively. Thus, the Excitation Function, $F_{\mathrm{EF}}$, and the Sensing Function, $F_{\mathrm{PUC}-\mathrm{SDC}}$, are the same: $F_{\mathrm{EF}}=3.38\cdot 10^{-3}\;\left(T\cdot \mathrm{Hz}\right)$ and $F_{\mathrm{PUC}-\mathrm{SDC}}=107,411{\;m}^{-1}$. The real part Transfer Function of the LIA, for sensitivity = 20 μV and cosθ=1, gives $F_{\mathrm{LIA}}^{/}=(1/\sqrt{2})(10/\mathrm{sensitivity})=353,553\;V$. Obviously, now both correction factors are $C_{p}=1$ (negligible porosity, thus nominal superconducting volume fraction 100%) and $C_{\mathrm{lr}}=1$ (negligible non-linear response, thus nominal linearly responding volume fraction 100%). Finally, the Response Function should be $F_{\mathrm{RF}}=V_{\mathrm{SC}}\left(-\langle \chi_{m,\mathrm{AC}}\left(T\right)\rangle \right)=\left(2.50\cdot 10^{-10}{\;m}^{3}\right)\left(14.70\right)=3.68\cdot 10^{-9}{\;m}^{3}$, where the *extrinsic* ACMS is given by $\langle \chi_{m,\mathrm{AC}}\left(T\right)\rangle =\langle \chi_{m,\mathrm{AC}}^{\mathrm{ext}}\left(T\right)\rangle =\langle \chi_{m,\mathrm{AC}}^{\mathrm{int}}\left(T\right)\rangle /\left(1-N\langle \chi_{m,\mathrm{AC}}^{\mathrm{int}}\left(T\right)\rangle \right)=-14.70$ when the single crystal is approximated by an orthogonal cuboid and $\langle \chi_{m,\mathrm{AC}}^{\mathrm{ext}}\left(T\right)\rangle =-15.55$ when the single crystal is approximated by a cylinder/disk. For these calculations, we used the fact that the *intrinsic* ACMS is $\langle \chi_{m,\mathrm{AC}}^{\mathrm{int}}\left(T\right)\rangle =-1$ (Meissner state) and the demagnetizing factor, N, is given by $N^{-1}=1+(3/4)(c/a)\left(1+\left(a/b\right)\right)=1/0.93$, for the case of the orthogonal cuboid with dimensions 2a= 1.59 mm, 2b= 1.88 mm and 2c= 84 μm, and $N^{-1}=1+1.6(c/a)=1/0.94$ for the case of the cylinder/disk with dimensions 2a= 1.95 mm and 2c= 84 μm [27,28]. Eventually, by recalling Relation (33), the theoretically expected signal should be $V_{\mathrm{DC}-\mathrm{the}}^{/}=-0.4713\;V$, or else the respective reduced signal should be $V_{\mathrm{DC}-\mathrm{the}}^{/}/\left(m\cdot B_{\mathrm{AC}}^{\mathrm{rms}}\right)=-0.5891\;V/\left(\mathrm{mg}\cdot G\right)$ for the case of the orthogonal cuboid, and $V_{\mathrm{DC}-\mathrm{the}}^{/}=-0.4983\;V$, or else the respective reduced signal should be $V_{\mathrm{DC}-\mathrm{the}}^{/}/\left(m\cdot B_{\mathrm{AC}}^{\mathrm{rms}}\right)=-0.6229\;V/\left(\mathrm{mg}\cdot G\right)$ for the case of the cylinder/disc where we used m=1.6 mg and $B_{\mathrm{AC}}^{\mathrm{rms}}=0.5\;G$. When these theoretically estimated levels of $V_{\mathrm{DC}-\mathrm{the}}^{/}/\left(m\cdot B_{\mathrm{AC}}^{\mathrm{rms}}\right)$ are determined for the two cases, the experimental data are superimposed for the sake of the presentation in Figure 5b. The comparison between the experimental, $V_{\mathrm{DC}-\exp}^{/}$, and the theoretical, $V_{\mathrm{DC}-\mathrm{the}}^{/}$, data evidences a percentage difference for the case of the orthogonal cuboid of

(38) $$\frac{V_{\mathrm{DC}-\mathrm{the}}^{/}-V_{\mathrm{DC}-\exp}^{/}}{V_{\mathrm{DC}-\exp}^{/}}100\%=6.8\%\;$$

while for the case of the cylinder/disk, it is

(39) $$\frac{V_{\mathrm{DC}-\mathrm{the}}^{/}-V_{\mathrm{DC}-\exp}^{/}}{V_{\mathrm{DC}-\exp}^{/}}100\%=12.9\%$$

We see that for the single crystal of YBa_2_Cu_3_O_7−δ_, which is *compact* (negligible porosity) and *linearly responding* (absence of grains/weak links), the agreement between the experimental and the theoretically expected data is, at least, fair. This validates our former expectation that the porosity and the non-linear response of the poly-crystalline cylindrical specimens of YBa_2_Cu_3_O_7−δ_ and Bi_2−x_Pb_x_Sr_2_Ca_2_Cu_3_O_10+y_ were responsible for the complexity in the theoretical modeling of the Response Function, $F_{\mathrm{RF}}$, and the relatively high percentage difference between the experimental and the theoretically expected data, discussed in the above subsections.

Still, we should comment on the fact that though the mathematical modeling of the ACMS was obtained for the case of an infinitely long cylinder, we employed those results (Relations (20)–(23)) for the realistic case of a cylinder/disc of finite height. Indeed, this is a serious assumption that we believe is justified by the use of the demagnetizing factor. From the fair consistency between the experimental and the theoretically expected data, we infer that the introduced demagnetizing factor captures the underlying physical processes that take place in the finite cylinder/disc quite effectively. In the same context, our results indicate that the odd shape of the single crystal and the particular shape approximation that is used (orthogonal cuboid or cylinder/disc) do not play any dramatic role. This seemingly weird behavior can be easily explained since it stems from the low-aspect-ratio height/diameter of the single crystal. Specifically, the single crystal has an effective height/diameter aspect ratio of 2c⁄2a= 84 μm / 1.59 mm ~ 0.053 when approximated by a plate/cuboid and 2c⁄2a= 84 μm / 1.95 mm ~ 0.043 when approximated by a cylinder/disc. In the results presented in Figure 7 of [27], we see that the distinct curves referring to different shapes clearly coincide in the limit of low aspect ratio values (high values, close to 1, of the demagnetizing factor). Thus, in this limit, the specific shape of the thin-plate specimen does not play any crucial role, as in our case.

Nevertheless, for all three cases investigated in this work, the overall outcome is very satisfactory when we consider that it relies on the straightforward comparison between the mathematical modeling of the entire experimental set-up with raw experimental data, without making any crude assumption/approximation during the algebraic part and without using any reference specimen/material to calibrate the ACMS unit.

Finally, we stress that the sensitivity/detection limit of the assembly of the four coaxial PUCs in the SDC is very high. We can calculate it from the raw experimental data presented in Figure 5 for the superconducting single crystal of YBa_2_Cu_3_O_7−δ_ that obviously exhibits perfect diamagnetism (Meissner state, $\chi_{m}=-1$). Simple algebra reveals that the sensitivity/detection limit of the particular PUCs in the SDC employed in our ACMS experimental set-up is greater than $1\;\mu V/\left(\mathrm{mg}\cdot G\right)$. This value is outstanding if one takes into account the highly demanding nature of this particular single crystal of quite low mass, m=1.6 mg.

## 6. Conclusions

The ACMS technique was explored theoretically, through detailed mathematical modeling, and experimentally, through investigation of representative specimens of poly-crystalline YBa_2_Cu_3_O_7−δ_ and Bi_2−x_Pb_x_Sr_2_Ca_2_Cu_3_O_10+y_ and single-crystalline YBa_2_Cu_3_O_7−δ_. Specifically, we calculated the DC voltage output signal in a closed form for a set-up based on four coaxial PUCs in the SDC. We clearly showed how the DC voltage output signal can be translated directly to the so-called *extrinsic* ACMS of a linearly responding superconducting specimen. From the latter, we draw the truly *intrinsic* ACMS of the parent material by taking into account the specific characteristics of the studied high-T_c_ specimens such as shape and dimensions for the demagnetizing effect and porosity for the estimation of the superconducting volume fraction. Thus, our mathematical model analytically takes into account all characteristics of the experimental hardware and of the studied linearly responding specimens so that our overall approach does not need any reference specimen/material to quantitatively calibrate the ACMS unit. The comparison of the mathematical model with experimental results obtained on bulk, poly-crystalline, cylindrical specimens of YBa_2_Cu_3_O_7−δ_ and Bi_2−x_Pb_x_Sr_2_Ca_2_Cu_3_O_10+y_ was not precise due to the porosity and the not entirely linearly responding nature of these specimens. On the contrary, the experimental results obtained on single-crystalline, thin-plate YBa_2_Cu_3_O_7−δ_, which is compact and linearly responding, were reproduced successfully. The overall modeling of the ACMS experimental set-up presented here is generic and, under certain conditions, can be used to obtain quantitatively reliable results on the *extrinsic*/*intrinsic* ACMS of any specimen/material.

## Data Availability

The data that support the findings of this study are available from the corresponding author upon reasonable request.
